# A previously unrecognized class of fungal ice-nucleating proteins with bacterial ancestry

**DOI:** 10.1126/sciadv.aed9652

**Published:** 2026-03-11

**Authors:** Rosemary J. Eufemio, Mariah Rojas, Kaden Shaw, Ingrid de Almeida Ribeiro, Hao-Bo Guo, Galit Renzer, Kassaye Belay, Haijie Liu, Parkesh Suseendran, Xiaofeng Wang, Janine Fröhlich-Nowoisky, Ulrich Pöschl, Mischa Bonn, Rajiv J. Berry, Valeria Molinero, Boris A. Vinatzer, Konrad Meister

**Affiliations:** ^1^ Department of Chemistry and Biochemistry, Boise State University, Boise, ID 83725, USA.; ^2^ School of Plant and Environmental Sciences, Virginia Tech, Blacksburg, VA 24061, USA.; ^3^ Department of Chemistry, The University of Utah, Salt Lake City, UT 84112, USA.; ^4^ Materials and Manufacturing Directorate, Air Force Research Laboratory, Wright-Patterson Air Force Base, Dayton, OH 45433, USA.; ^5^ Department of Molecular Spectroscopy, Max Planck Institute for Polymer Research, 55128 Mainz, Germany.; ^6^ Department of Multiphase Chemistry, Max Planck Institute for Chemistry, Mainz 55128, Germany.

## Abstract

Ice-nucleating proteins (INpros) catalyze ice formation at high subzero temperatures, with major biological and environmental implications. While bacterial INpros have been structurally characterized, their counterparts in other organisms have remained largely unknown. Here, we identify membrane-independent proteins in fungi of the *Mortierellaceae* family that promote ice formation with high efficiency. These proteins are predicted to adopt β-solenoid folds and multimerize to form extended ice-binding surfaces, exhibiting mechanistic parallels with bacterial INpros. Structural modeling, phylogenetic analysis, and heterologous gene expression leading to ice nucleation in *Escherichia coli* and *Saccharomyces cerevisiae* show that the fungal INpros are encoded by orthologs of the bacterial *InaZ* gene, which was likely acquired by a fungal ancestor through horizontal gene transfer. The discovery of cell-free fungal INpros provides tools for innovative freezing applications and reveals biophysical constraints on ice nucleation across life.

## Teaser

Discovery of potent soluble fungal ice nucleators uncovers a conserved molecular approach to promoting ice formation.

## INTRODUCTION

The crystallization of water is the most ubiquitous phase transition on Earth, influencing ecological interactions, atmospheric processes, and climate dynamics. Ice formation is thermodynamically favorable below 0°C, but it is kinetically hindered because of the high energetic cost of forming an initial ice nucleus (*1*). Above −46°C, homogeneous ice nucleation is very slow (*2*), and various substrates can act as catalysts to heterogeneously accelerate ice nucleation. In nature, the freezing of water is facilitated by ice nucleators (INs) of biological and abiotic origins. Biological INs are found across all kingdoms of life (*3*–*8*), with those from bacteria and fungi being the most efficient, enabling ice formation at temperatures as high as −2°C (*9*, *10*). In contrast, most abiotic INs freeze water at lower temperatures (*11*, *12*), while silver iodide, a widely used artificial cloud-seeding agent, initiates ice formation around −5°C (*13*). Efficient biological INs can affect crop frost damage, cloud formation, and precipitation, and their unmatched efficiencies enabled applications in artificial snowmaking and cryopreservation (*14*, *15*). Despite the diversity of biological INs capable of nucleating ice above −10°C, only bacterial INs have been fully sequenced and structurally characterized, serving as the key model for understanding biomolecular ice formation. The ice-nucleating activity of Gammaproteobacteria like *Pseudomonas syringae* originates from a highly conserved family of membrane-associated ice-nucleating proteins (INpros) encoded by the *Ina*Z gene (*16*). The InaZ protein consists of ~1200 residues and has a three-domain architecture, with an N-terminal membrane-anchoring domain, a central repeating domain (CRD), and a C-terminal capping domain. The CRD, which constitutes most of the structure, comprises 50 to 100 tandem repeats of a conserved 16-residue sequence and adopts a β-solenoid fold. This rigid, repetitive architecture serves as a molecular scaffold for ice nucleation, with the conserved motifs threonine-X-threonine (TxT) and serine-leucine-threonine (SLT) facilitating the ordering of interfacial water molecules to promote ice formation (*17*). The CRD further enables the assembly of bacterial INpros into large functional aggregates, where ice-nucleating efficiency depends on the size and spatial organization of nucleation sites. This supramolecular organization relies on an intact membrane (*18*–*20*), as individual INpros lack the templating area required to nucleate ice at temperatures close to 0°C (*18*, *21*, *22*). Unlike bacterial INpros, the genetic and molecular basis of ice formation in other organisms is largely unknown. However, several widely distributed fungi and lichen species exhibit highly efficient and exceptionally stable ice-nucleating activity (*6*, *7*, *10*, *23*) that enables freezing above −5°C. These INs function independent of cell membranes (*23*), withstand extreme pH conditions (2 to 12) (*24*), and have been confirmed as proteinaceous through circular dichroism and their sensitivity to proteolytic digestion and heat denaturation (*10*, *25*). Size characterization experiments suggest that they are smaller than bacterial INpros and nucleate ice through a fundamentally distinct, nonmembrane-bound mechanism (*6*, *10*, *25*). Here, we examine the genetic basis, composition, and structure of these potent biological INs to elucidate the molecular determinants of their size, stability, and supramolecular aggregation mechanisms that drive their remarkable ice-nucleating activity.

## RESULTS

### Genome sequencing reveals orthologs of bacterial *InaZ* in fungi

We sequenced the genomes of the ice nucleation active strain 13A of *Mortierella alpina* (*23*) and a pure fungal culture, L01-tf-B03, obtained from the lichen *Peltigera britannica* (*26*). DNA extracted from these ice nucleation active–confirmed strains was sequenced using Illumina technology, with sequencing reads assembled into high-quality draft genomes. The genome sequences were annotated, with gene predictions informed by genomes of related species. For the *M. alpina* genome, the most closely related, publicly available genomes were those of other *M. alpina* strains, as determined by sequence similarity in the internal transcribed spacer (ITS) locus. Unexpectedly, the fungal culture derived from *P. britannica* also exhibited the highest sequence similarity to species within the family *Mortierellaceae*. A core-genome tree using genes that were present in single copy (*n* = 224) in all members of the family was then constructed (fig. S1) and confirmed the identity of the *M. alpina* 13A strain based on its location within a clade consisting of other *M. alpina* strains. The fungal culture L01-tf-B03 from *P. britannica* shared a common ancestor with two *Entomortierella parvispora* strains, suggesting L01-tf-B03 to be affiliated with that species.

**Fig. 1.**
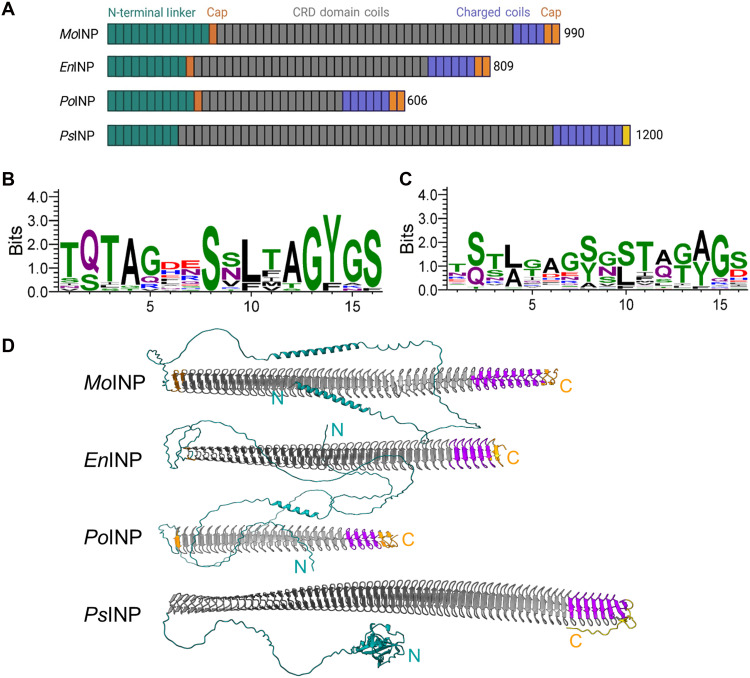
Comparative analysis of fungal and bacterial INpros. (**A**) Schematic domain map of *Mo*INpro, *En*INpro, *PPo*INpro, and *Ps*INpro, highlighting conserved and variable regions. (**B** and **C**) WebLogo plots of the first 33 and the last 7 16-residue repeats in *En*INpro CRD, illustrating sequence conservation. The overall stack height reflects the degree of conservation, while individual letter heights represent the relative frequency of each amino acid at that position. (**D**) AlphaFold3 models of *Mo*INpro, *En*INpro, *Po*INpro, and *Ps*INpro with the characteristic β strands of the CRD. The models are colored by different domains and correspond to those shown in (A).

Next, we screened the sequences for INpros by searching for conserved regions and known ice-binding motifs, such as TxT and SLT. Using this approach, we identified two candidate INpro sequences, which we analyzed for similarity to bacterial INpros. We performed a basic local alignment search to identify regions of sequence similarity at the protein level between the fungal INpros, known bacterial INpros, and other potential INpros. Using this approach, we identified a third potential INpro with high sequence similarity from *Podila clonocystis*, also in the *Mortierellaceae* family (*27*). Figure 1A presents the comparative analysis of fungal and bacterial INpro sequences. We found that the INpros from *M. alpina* (*Mo*INpro), *E. parvispora* (*En*INpro), and *P. clonocystis* (*Po*INpro) consist of 990 (100 kDa), 809 (81.8 kDa), and 606 (64 kDa) amino acids, respectively. These fungal INpros are smaller than the 120-kDa INpros from *P. syringae* (*Ps*INpro).

Multiple sequence alignments reveal that fungal INpros share high sequence similarity with each other (~75%) and with bacterial *InaZ* proteins (~58%) despite the differences in size (figs. S3 and S4). For *P. syringae*, the *Ps*INpro gene encodes a long β-solenoid fold, comprising a CRD with 51 coils and 10 charge-rich coils, each consisting of 16 residues (Fig. 1A). *Ps*INpro further features a short C-terminal region and a long, flexible N-terminal domain. The length, location, and sequence of charged residues and the CRD–to–charged-coil ratio are highly conserved across bacterial INpros and likely critical for INpro multimerization (*28*). The largest sequence overlap between fungal and bacterial INpros occurs in the CRD, with nearly 100% similarity. This region contains repetitive TxT and SxLT motifs (Fig. 1B), which serve as putative ice-nucleating motifs (*17*, *21*). The other highly conserved tyrosine-glycine-serine (YGS) motifs are likely not involved in ice binding but rather provide an INpro dimerization interface (*18*, *22*, *29*). The fungal INpros also have flexible N-terminal domains of varying lengths but lack a defined membrane-anchoring region. The charge-rich coils near the C terminus exhibit fewer repetitive motifs, and the alignment of annotated INpro gene regions reveals lower sequence conservation between fungi and bacteria (Fig. 1 and fig. S3). All fungal INpros contain six cysteine residues near the C terminus and two cysteine residues at the start of the CRD domain, which are capable of disulfide bond formation. These features are unique to fungal INpros, likely playing key roles in their structural arrangement and stability.

**Fig. 2.**
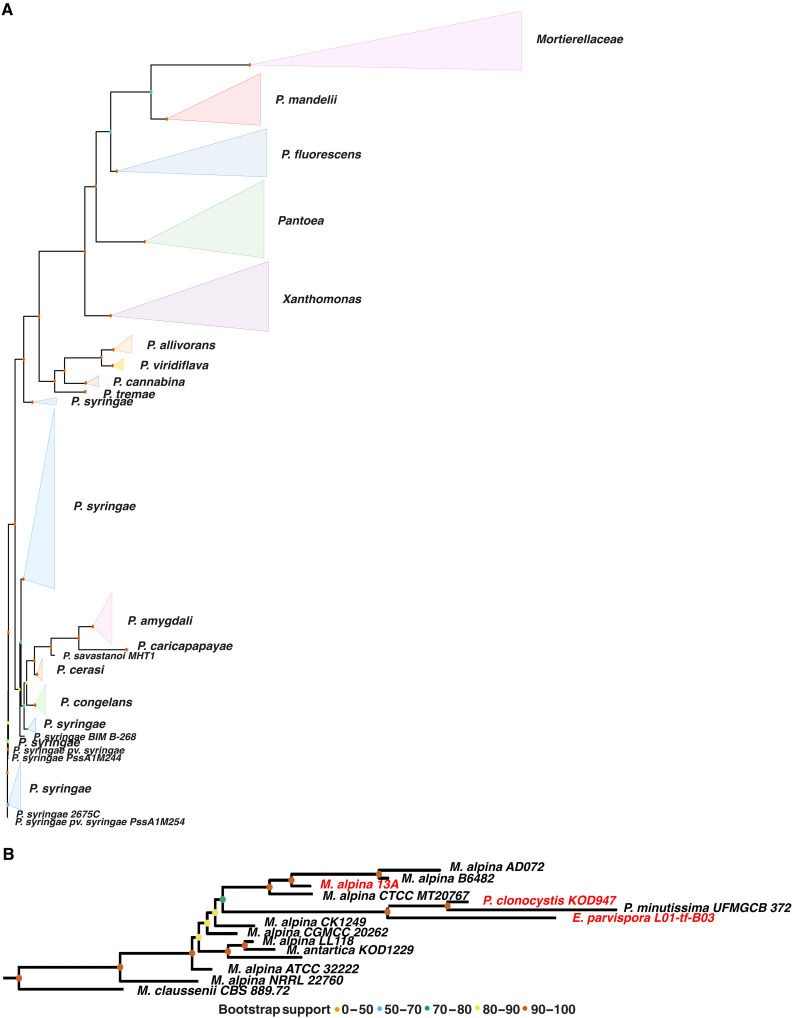
Phylogenetic relationship between fungal and bacterial INpro genes. (**A**) Maximum likelihood phylogenetic tree illustrating the evolutionary placement of the genes coding for INpros in the *Mortierellaceae* relative to bacterial *InaZ* orthologs from *Pantoea*, *Pseudomonas*, and *Xanthomonas.* (**B**) The *Mortierellaceae* clade from the maximum likelihood tree with the genes encoding the INpros shown in Fig. 1 highlighted in red. Bootstrap values are shown as colored circles at the nodes.

Given the protein similarities, we performed a phylogenetic analysis to compare the DNA sequences of bacterial *InaZ* genes and the genes encoding the fungal INpro to determine whether these similarities arose through convergent evolution or horizontal gene transfer (HGT). Figure 2 presents a phylogenetic tree of 218 *InaZ* orthologs in the bacterial genera *Pantoea*, *Pseudomonas*, and *Xanthomonas* and 15 InaZ orthologs identified in the *Mortierellaceae* (encoding the three proteins described above and 12 orthologs found in additional *Mortierellaceae* genome sequences; table S1). While most bacterial clades are collapsed in Fig. 2, fig. S2 shows the tree with all 217 individual genes. The 15 INpro genes from the *Mortierellaceae* family form a single cluster that shares a most recent common ancestor (MRCA) with *InaZ* orthologs of *Pseudomonas mandelii*. However, the bootstrap support for the MRCA is only 61%, while the first node with a high support is the node representing the MRCA of the *Mortierellaceae*, *P. mandelii*, *Pseudomonas fluorescens*, and *Pantoea InaZ* sequences. This suggests that the *InaZ* gene was originally acquired by an ancient *Mortierellaceae* strain through an HGT event from an ancient bacterial strain that had an *InaZ* gene sequence ancestral to today’s *P. mandelii*, *P. fluorescens*, and *Pantoea InaZ* sequences. The bacterial origin of the fungal *InaZ* orthologs is further supported by their guanine (G) and cytosine (C) nucleotides (GC) content, which is higher compared to a set of randomly selected fungal genes and more similar to the GC content of bacterial genes, including bacterial *InaZ* genes (fig. S3). Codon usage of the fungal *InaZ* orthologs is distinct from that of both other fungal genes and bacterial genes (fig. S3), while codon usage of bacterial *InaZ* genes is indistinguishable from other bacterial genes. These results support a bacterial origin of the fungal *InaZ* orthologs whereby GC content and codon usage became more similar to the overall GC content and codon usage of the recipient fungal genomes over evolutionary times, a well-studied evolutionary mechanism (*30*–*32*). The fungal INpros also exhibit unique features that are entirely absent in bacterial INpros. These findings suggest that while the DNA fragment encoding the core INpro framework may have been acquired by HGT from bacteria, subsequent evolutionary adaptations, such as a merger with a fungal gene, optimized function in fungi.

### Genetic confirmation of fungal *InaZ* orthologs as encoding INpro

**Fig. 3.**
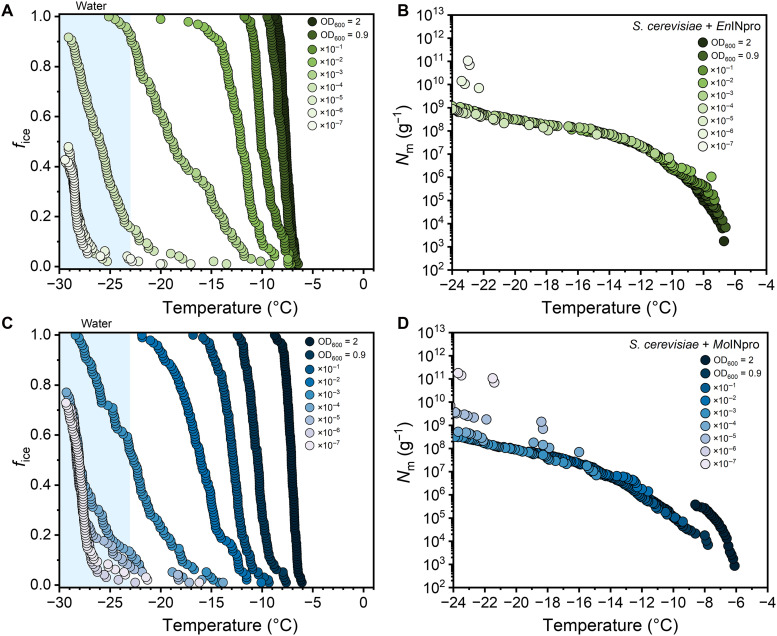
Ice nucleation activity of yeast strains expressing fungal INpros. (**A**) Fraction of ice (*f*_ice_) and cumulative freezing spectra (**B**) for a serial dilution of *S. cerevisiae* expressing *En*INpro. The presented *f*_ice_ data correspond to the *N*_m_ plot shown in (B). (**C**) Fraction of ice (*f*_ice_) and cumulative freezing spectra (**D**) for a serial dilution of *S. cerevisiae* expressing *Mo*INpro. The presented *f*_ice_ data correspond to the *N*_m_ plot shown in (C). Serial 10-fold dilutions were prepared from IN samples with optical densities of OD_600_ = 0.9 at 600 nm, which corresponds to a concentration of ~1 mg/ml. Blue-shaded regions represent the freezing range of pure water in our setup (<−23°C).

To confirm that the proteins encoded by the fungal orthologs of the bacterial *InaZ* gene do have ice nucleation activity, plasmids carrying the *InaZ* orthologs from *M. alpina* 13A (*Mo*INpro) and *E. parvispora* L01-tf-B03 (*En*INpro) were constructed and introduced in *Saccharomyces cerevisiae* and *Escherichia coli*. No freezing events were observed in the negative controls: water or the control strains (*S. cerevisiae* and *E. coli*) not expressing *InaZ* orthologs (fig. S6). In contrast, Fig. 3 shows that heterologous expression of *Mo*INpro and *En*INpro in *S. cerevisiae* is sufficient to confer potent ice nucleation activity. For *Mo*INpro, expression in yeast shifts the nucleation from −26°C to *T*_50_ ~ −7.1°C, while expression in *E. coli* shifts it to *T*_50_ ~ −14.4°C (fig. S6). For *En*INpro, expression in yeast results in a shift to *T*_50_ ~ −7.4°C and, in *E. coli*, to *T*_50_ ~ −15.3°C (fig. S6). *T*_50_ is defined as the temperature at which 50% of droplets in the assay have frozen. These results establish that the identified genes encode active INpros. Dilution series and cumulative freezing spectra (Fig. 3B) confirm that this gain of function is unambiguous and, in the case of *En*INpro, resembles the activity of the native host within ±1°C (fig. S7). The activity profiles of *Mo*INpro and *En*INpro expressed in *E. coli* do not reproduce the profiles observed in the native fungi. Such differences are expected when proteins are expressed outside their native host, where differences in pH and ionic conditions (*33*), membrane composition, and organism-specific cofactors can affect protein assembly (*34*). Consistent with this, previous studies have shown that heterologous expression of bacterial *InaZ* in yeast (*35*) and plant cells (*36*) reduced activity compared to native bacteria, whereas expression in closely related Gram-negative bacteria yields activities that more closely match those of the native bacteria (*17*, *22*). Yet, only deleting the *InaZ* ortholog in a *Mortierellaceae* strain and observing a loss of ice nucleation activity would formally prove that the *InaZ* orthologs not only encode INpros but are also the sole genes required for ice nucleation activity in native *Mortierellaceae.* However, given the high sequence similarity between fungal and bacterial *InaZ* genes and the demonstrated sufficiency of fungal *InaZ* genes in conferring ice nucleation activity to bacteria and yeast, it is highly unlikely that different, or additional, genes are required for this function in *Mortierellaceae*.

### Structure of *Mo*INpro, *En*INpro, and *Po*INpro predicted by AlphaFold3

**Fig. 4.**
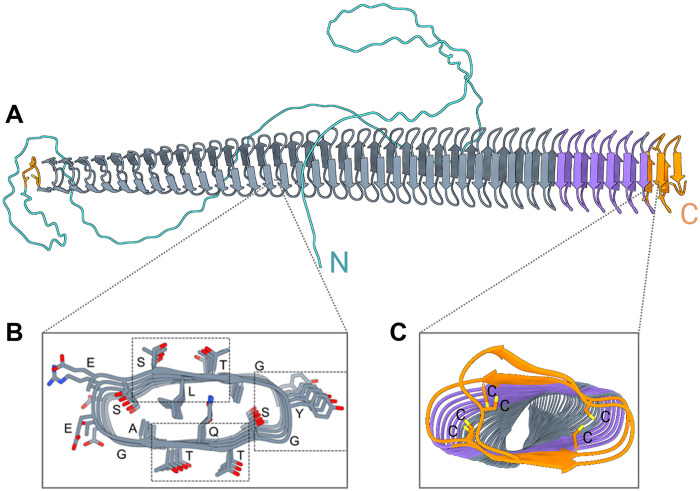
AlphaFold3 model of *En*INpro and cross sections through the solenoid. (**A**) The model of *En*INpro is colored by different domains with arrows representing β strands. The model highlights a β-solenoid fold (gray, purple) adjacent to the disulfide capping motif (orange). (**B**) Cross section through the central domain region. Residues are identified by their one-letter codes. Boxes indicated the location of the characteristic TQT, SLT, and YGS motifs. (**C**) Cross section of the central domain’s capping structure, where adjacent cysteine residues can form disulfide bonds that likely stabilize the β-solenoid fold.

Figure 4 shows the AlphaFold3 model of *En*INpro from *E. parvispora* (*37*). The structure consists of an N-terminal domain with a 158-residue flexible linker, followed by a central repetitive domain of ~19 nm, modeled as a continuous β-solenoid. Within the β-solenoid fold, each 16-residue repeat forms a single coil, and the overall fold closely resembles the previously characterized bacterial INpro β-solenoids. The β-solenoid can be divided into two distinct regions. The first region consists of 20 coils with a highly repetitive sequence, containing conserved TxT, SLT, and YGS motifs. These motifs form long parallel arrays that are likely responsible for the ice-nucleating function of the fungal protein (*17*, *21*), and the cross-sectional geometry of the motifs is identical to that found in bacterial INpros. The second region forms a transition zone, where the repetitive sequence becomes less conserved. This region consists of seven coils, includes the C-terminal domain, and lacks repetitive sequence features. Instead, it contains positively and negatively charged amino acids distributed in patches (figs. S8 and S9) likely assisting in the intermolecular assembly of the INpros (*17*, *18*). Moreover, the region contains six cysteine residues that are capable of forming disulfide bonds. An additional pair of cysteines is located near the beginning of the β-solenoid fold. Disulfide bonds are known to stabilize antifreeze protein solenoids (*38*), and in *En*INpros, they may also function as structural caps of the β-solenoid, preserving the fold and preventing end-to-end associations. This stabilization mechanism clearly differentiates fungal INpros from bacterial INpros, where the C-terminal region is enriched in arginine residues and contains a 41-residue C-terminal cap structure, while the N-terminal cap is unknown. Over the 28 repeats, the *En*INpro model predicts a slight left-handed twist in the solenoid, similar to bacterial INpros. The slight rotation between adjacent coils will likely be eliminated as neighboring solenoids dimerize, forming a large flat surface required for efficient ice nucleation (*21*).

In *Ps*INpro, positively charged lysine and arginine residues are clustering near the C terminus, while negatively charged aspartic and glutamic acid residues are localized near the N terminus, all positioned on one side of the solenoid. In contrast, *En*INpros show a more evenly distributed charge pattern with alternating positive and negative residues rather than distinct clusters. In addition, fungal INpros feature a highly charged N-terminal flexible linker, characterized by distinct patches of positive and negative charge, further distinguishing them from bacterial INpros. The fungal INpros also incorporate histidine residues, which are largely absent in bacterial INpros. Overall, the differences in electrostatic surface properties are likely to influence INpro multimerization and stabilization.

Next, we modeled the protein structures of putative INpros from additional ice nucleation active fungal strains (figs. S10 and S11). The *Mo*INpro and *Po*INpro exhibit structural characteristics similar to the *En*INpro, differing primarily in the length of the solenoids and composition of the N-terminal flexible linker. *Mo*INpro, *En*INpro, and *Po*INpro have solenoid lengths of 25.3, 19, and 13.5 nm, and their CRD regions comprise 42, 31, and 23 repeats, respectively. All the fungal INpros further share a molecular width of 3.4 nm.

### Multimerization enables high fungal ice nucleation activity

**Fig. 5.**
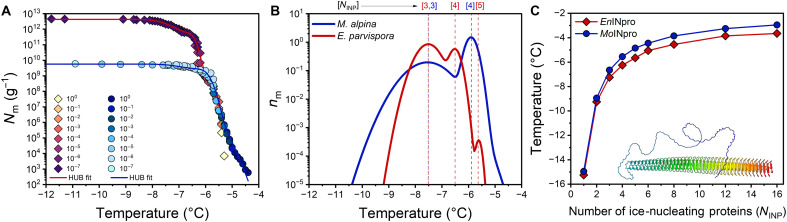
Freezing experiments with aqueous samples of fungal INs from *M. alpina* and *E. parvispora*. (**A**) Cumulative freezing spectra (number of INs per unit mass, *N*_m_) measured for *M. alpina* and *E. parvispora*. The lines represent the optimized solution obtained using the HUB-backward code, assuming that the differential spectra consist of two Gaussian subpopulations for *M. alpina* (blue line) and three Gaussian subpopulations for *E. parvispora* (dark red line) as shown in the next panel. (**B**) Differential freezing spectra [normalized distribution function, *n*_m_ (*T*)] derived from (A) exhibiting modal freezing temperatures of −5.9° and −7.5°C for *Mo*INpros and −5.6°, −6.5°, and −7.5°C for *En*INpros (tables S2 and S3). (**C**) Ice nucleation temperatures predicted as a function of the number of aggregated *Mo*INpro and *En*INpro based on CNT as implemented in the HINT algorithm (table S4). Blue circles and red squares indicate the freezing temperatures of rectangular surfaces formed by parallel-aligned INpro monomers, assuming an ice-binding surface of 3.4-nm width and a length of 25.3 nm for *Mo*INpro (blue) and 19 nm for *En*INpro (red), based on AlphaFold3 structural predictions.

Figure 5A shows the results of ice nucleation experiments with mycelial washes from the ice nucleation active strains *M. alpina* and *E. parvispora*. The cumulative freezing spectra comprise a complete dilution series with initial mycelial wash concentrations of ~1 mg/ml. They exhibit two distinct increases in the cumulative number of ice nuclei per unit mass, *N*_m_ (*T*), for *E. parvispora* at ~−5.6° and ~−6.5°C, while *M. alpina* exhibits a single rise at ~−4.8°C, followed by a plateau below −8°C, confirming that the investigated fungal INs are highly efficient (*23*, *39*). The corresponding differential freezing spectra in Fig. 5B show the ice nucleation temperature distributions for *M. alpina* and *E. parvispora* derived from the experimental *N*_m_ (*T*) dataset using the heterogeneous underlying-based (HUB) backward code (*40*), which fits the experimental *N*_m_ (*T*) data of droplet freezing experiments by a linear combination of Gaussian subpopulations (table S2). The best-fit differential spectra exhibit two Gaussian subpopulations centered at −5.9° and −7.5°C for *M. alpina* and three subpopulations at −5.6°, −6.5°, and −7.5°C for *E. parvispora*.

The INpros provide a surface that can bind and order water molecules, thereby reducing the free energy barrier for ice nucleation. To estimate the number of INpros required to form aggregates capable of promoting ice nucleation at the observed freezing temperatures, we applied the Heterogeneous Ice Nucleation Temperature (HINT) algorithm (*21*), which uses a numerical implementation of classical nucleation theory (CNT) to determine the free energy barriers associated with ice formation and growth on finite surfaces. Leveraging experimentally derived thermodynamic properties of water, HINT enables calculations of the heterogeneous freezing temperature as a function of surface size. Simulations by Qiu *et al.* (*21*) have shown that increasing the length of the ice-binding β helix in bacterial INpros does not substantially affect freezing efficiency, as these proteins are already approximately eight times longer than they are wide. In contrast, increasing surface width through parallel alignment of INpros markedly improves nucleation efficiency by generating a more square-like ice-binding interface. Given that fungal INpros also adopt elongated β-helical architectures, this suggests that warmer nucleation temperatures are governed primarily by surface width and higher-order assembly rather than protein length alone (*21*). In our calculations, we assumed that the ice-binding surface is flat, that the distance between monomers remains constant, and that the monomer size is characterized by the AlphaFold3 parameters of the solenoid (length: 25.3 nm *Mo*INpro, 19 nm *En*INpro; width 3.4 nm). Figure 5C shows the ice nucleation temperatures predicted for side-by-side INpro aggregates of *Mo*INpro and *En*INpro. This comparison suggests that, for *E. parvispora* and *M. alpina*, the freezing modes that exhibit lower nucleation efficiency (−7.5°C) originate from INpro trimers, whereas the more efficient ice-nucleating structures (−6.5° and −5.9°C, respectively) correspond to INpro tetramers. The highest freezing mode of *E. parvispora* at −5.6°C can be attributed to the formation of INpro pentamers.

This finding raises the question of what governs the aggregation and ice-nucleating activity of fungal INpro multimers. We propose that multimerization is driven by stacking of highly conserved tyrosine (Tyr) ladders, a process also suggested for bacterial INpros (*18*, *22*, *29*). The Tyr ladders create a structurally aligned surface, orienting ice-nucleating motifs in parallel and expanding the effective nucleation site. Further aggregation and multimer formation are likely mediated by electrostatic interactions (*18*). The electrostatic surface models (figs. S6, S7, and S13) of the fungal INpros reveal distinct charge distributions along the solenoid and the flexible N-terminal linker, suggesting that multimerization arises from attraction between oppositely charged residues and may be stabilized by the N-terminal linker, enhancing structural integrity and assembly efficiency. A schematic representation of this proposed mechanism is provided in fig. S13.

The function of fungal INpros independent of the membrane is supported by several independent lines of evidence; first, the fungal INpro sequence of *M. alpina* contains a canonical N-terminal signal peptide, consistent with secretion into the extracellular environment rather than membrane anchoring. In line with this, ice-nucleating activity was recovered from aqueous solution following washing of mycelia. Second, ice-nucleating activity persists after sequential filtration through 5-μm, 0.1-μm, and 300-kDa cutoffs, yielding freezing spectra essentially indistinguishable from the original sample (*6*, *23*). These steps remove intact cells, large debris, and membrane vesicles, consistent with membrane-independent ice-nucleating activity. In addition, the activity is resistant to treatments that perturb membranes, including lipases, surfactants, and membrane fluidizers, as well as to repeated freeze-thaw cycles and elevated temperatures (figs. S12 and S14). This behavior contrasts with bacterial INpros, whose highest activities depend strongly on membrane association and are sensitive to membrane perturbation.

Freezing spectra of purified fungal INpros (fig. S16) further demonstrate that the INpros alone are sufficient to enable high ice nucleation activity, independent of cellular or membrane components. Obtaining atomistic structural information on the fungal INpros and their functional assemblies during ice formation remains an important goal for future work. At present, these approaches are limited by the availability of purified fungal INpros, which can now be obtained only at nanomolar concentrations and are thus not amenable to high-resolution structural characterization.

## DISCUSSION

The discovery of soluble fungal INpros extends our understanding of how organisms overcome the kinetic barriers of ice formation. Despite their distant taxonomic origins, fungal and bacterial INpros share a conserved β-solenoid architecture and multimerization strategy, hallmarks of highly efficient ice nucleation (*10*).

However, fungal INpros function independently of membrane attachment and likely achieve stability through a distinct disulfide capping mechanism (*17*, *38*). Structural details into INpros are now obtained through artificial intelligence (AI)–based structure predictions (*22*, *41*); atomic resolution experiments are still needed to confirm INpro structures and assembly mechanism. Our proposed aggregation mechanism (fig. S13) combines the AI-predicted INpro structure with experimentally parameterized CNT for finite shapes, interpreting the observed freezing spectra. This provides strong support for the proposed stoichiometry and INpro assembly; however, further experimental work is needed to resolve the molecular mechanism, including whether cofactors influence the formation of functional aggregates.

Our findings suggest that HGT enabled *Mortierellaceae* fungi to co-opt a bacterial ice nucleation framework, which was subsequently adapted for soluble, cell-free activity. This convergence underscores fundamental physical constraints on protein-mediated ice nucleation while revealing the versatility of biological solutions to environmental challenges. In addition to *Mortierellaceae* fungi, we also sequenced and analyzed genomes of two ice nucleation active *Fusarium* strains (*10*, *25*) but found no proteins related to the INpros described here. This is consistent with previous studies that proposed that ice nucleation in *Fusarium* is enabled by much smaller proteins (*10*, *25*).

Beyond their biological significance, the fungal INpros exhibit extraordinary stability and potency, functioning at picomolar concentrations and under extreme conditions (figs. S14 and S15). Their membrane independence and solubility clearly distinguish them from bacterial INpros and could unlock possibilities for bioinspired freezing technologies, from precision cryopreservation to engineered weather modulation. The identification of such proteins in common soil fungi further raises the possibility that their contribution to atmospheric ice nucleation is now underestimated (*23*, *42*). The fungal INpros’ ability to remain highly active at low concentrations and under harsh conditions (figs. S14 and S15) may allow them to retain function if aerosolized. Future work should focus on quantifying their environmental prevalence and exploring their potential in synthetic and applied contexts. More broadly, this study shows how gene transfer and structural convergence can drive evolutionary innovation at the interface of biology and physics.

## MATERIALS AND METHODS

### Genome sequencing

Strains were grown on potato dextrose agar or malt yeast extract agar (Difco, Thermo Fisher Scientific, USA) for 2 to 4 weeks at room temperature (*23*, *43*), and the mycelium was harvested using an ethanol-sterilized razor blade. DNA was extracted using the QIAGEN All Prep Fungal DNA/RNA/Protein Kit (Hilden, Germany). DNA was eluted in molecular-grade water and stored at −80°C until sequencing.

Short-read sequencing was done on an Illumina NovaSeq X sequencer by SeqCenter (Pittsburgh, PA). Quality control and adapter trimming were done by the company. Genome assembly was done using SPAdes version 3.15.5. The assembled genomes were then annotated using Augustus version 3.5.0 using *Fusarium graminearum* as the model (*44*, *45*).

### Core genome phylogeny of the *Mortierellaceae*

To identify the genus and species of the isolates, the ITS region (ITS1, 5.8 S, and ITS2) was extracted using ITSx version 1.13 (*46*). The ITS region was blasted on National Center for Biotechnology Information (NCBI) and was found to be most closely associated with *Mortierellaceae* sequences. *Mortierellaceae* genomes used in a previous study were used to identify the specific genus and species of the isolates (*27*). The whole-genome assemblies of fungal genomes were downloaded from the NCBI Genome Assembly database on 24 July 2024, using the ncbi-datasets-cli tool version 14.2.2 (*47*). To construct a core genome tree, we used a combination of tools: Benchmarking Universal Single-Copy Orthologs version 5.8.2 to identify single-copy genes, Multiple Sequence Comparison by Log-Expectation (MUSCLE) version 5.3 to align the identified single-copy genes (*48*), and TrimAI version 1.5 to remove poorly aligned sequences (*49*). The core phylogenetic trees were constructed using IQ-TREE version 2.3 with maximum likelihood and ultrafast bootstrapping (*50*). Consensus trees were visualized using ggtree on R version 4.3.3 (Angel Food Cake) (*51*, *52*). *Linnemannia hyalina* and *Mortierella hygrophila* were used as outgroups.

### Phylogenetic analysis of bacterial and fungal *InaZ* orthologs

Several gene sequences associated with ice nucleation activity were downloaded from UniProt on 10 February 2025. *InaZ* from *P. syringae* (CAA26837.1) was used as a query sequence against a custom database of all available genome assemblies of *Mortierellaceae* and Gammaproteobacteria assemblies in NCBI. To construct the database of Gammaproteobacteria, we began by filtering all Gammaproteobacteria genomes available in NCBI. Following this initial selection, we used a threshold of 99% average nucleotide identity to identify representative strains within the dataset. To identify potential orthologs, 50% identity, 200 alignment length, and an *e*-value ≤ 1e^−5^ were used to filter results. Overlapping sequences found on the same contig were merged using the bedtools merge version 2.31.1 function (*53*). Only the best hit (highest percent identity) was retained for each assembly (table S1) and was used for phylogenetic analysis. Potential orthologs were then aligned using MUSCLE version 5.3, and TrimAl version 1.5 was used to remove poorly aligned sequences (*49*, *50*). The phylogenetic tree was constructed using IQTree version 2.3 with maximum likelihood and ultrafast bootstrapping (*51*). The predicted root for the tree was found by calculating bootstrap values with IQTREE using nonreversible models using the --model-joint UNREST flag (*54*). Trees were visualized using ggtree on R version 4.3.3 (Angel Food Cake).

Nucleotide composition and relative synonymous codon usage values were calculated for nine INpro genes and nine randomly selected coding sequences from Gammaproteobacteria and *Mortierellaceae* genomes using CAICal version 1.3 (*55*). GenBank data for the selected genomes were downloaded on 19 July 2025. Random coding sequences were chosen by generating random integers corresponding to gene order in the genome annotation; the genes matching these numbers were included in the analysis. All statistical analyses were performed in R version 4.3.3, and figures were generated using the ggplot2 package.

### Cloning of fungal InaZ orthologs in *S. cerevisiae* and *E. coli* and confirmation of ice nucleation activity

To clone INpro genes into a yeast vector, the *M. alpina Mo*INpro (g9171) coding sequence was amplified with sense primer VTXW3636 (CTATACTTTAACTGCAGTTTAATTA**ATGCGTCTCTTCCTCTCAA**), bold sequences are from *M. alpina* 13A, and antisense primer VTXW3637 (GCGAAGAAGTCCAAAGCTGGATCC**TCATTCATTATTACCACACC**). The *E. parvispora* L01-tf-B03 *En*INpro (g6506) coding sequence was amplified with sense primer XW3642 (CTATACTTTAACTGCAGTTTAATTA**ATGGGCACCGCTGATGTT**), bold sequences are from *E. parvispora*, and antisense primer VTXW4643 (GCGAAGAAGTCCAAAGCTGGATCC**TTATTGTCCACACTGGCC**) by using PrimeSTAR Max DNA polymerase (Takara Bio, CA) in an epGradient Mastercycler (Eppendorf, CT) as below: 98°C for 10 s to denature DNA templates; 30 cycles with the following settings: 98°C for 10 s; 55°C for 10 s; and 68°C for 30 s, followed by a 5-min extension at 68°C. Polymerase chain reaction (PCR) products were separated using agarose gel electrophoresis and purified using the AccuPrep PCR/Gel purification kit (Bioneer, CA). The gel-purified PCR fragments were cloned into a *Pac*I and *Xho*I-digested pB1YT3-FLAG-HA plasmid using the Genbuilder kit (GenScript, NJ) to make pU33-Ma_g9171 and pU33-EP_g6506. Both FLAG and HA tags were removed during cloning, and therefore, authentic proteins will be produced in yeast cells upon induction. After sequencing, plasmid DNA was transformed into yeast strain YPH500 (MAT-alpha, *ura*3-52 *lys*2-801 *ada*2-101 *trp*1-63 *his*3-200 *leu*2-1) by the lithium acetate method (*56*). After transformation, yeast cells were plated onto synthetic complete (SC) medium with 2% glucose as a carbon source but lacking uracil (SC-U) to maintain a selection for the plasmid. To assess ice nucleation activity, yeast cells harboring pU33-Mag9171 or pU33-EPg6506 or yeast cells harboring pRS416 as the negative control were streaked onto SC-U plates containing 2% galactose and incubated at 30°C for ≥36 hours to induce gene expression. For expression in bacteria, the INpro gene sequences of *E. parvispora* (g6506) and *M. alpina* (g9171) were synthesized and subcloned with a His-SUMO tag into the expression vector pET-16b by GenScript (GenScript, the Netherlands). The resulting plasmids were transformed into ClearColi electrocompetent cells (Biosearch Technologies, Germany) by electroporation. ClearColi cells are a genetically modified *E. coli* strain designed to reduce endotoxin contaminations in protein preparations. pUC19 DNA (Biosearch Technologies, Germany) and endotoxin-free water served as positive and negative transformation controls, respectively. After transformation, bacterial cells were plated onto LB-Miller agar with ampicillin (100 μg/ml) and grown at 37°C for 24 hours. Single colonies visible after 24 hours were transferred to 5-ml loose-lid tubes of LB-Miller medium with ampicillin (100 μg/ml) and incubated overnight at 37°C with 200 rpm shaking. The following day, 250 ml of prewarmed LB-Miller medium with ampicillin and 2% glucose [from 40% (w/v) autoclaved glucose] was inoculated with 4.2 ml of the overnight culture and grown at 37°C with 200 rpm shaking until an OD_600_ (optical density at 600 nm) of 0.6 was reached. Gene expression was induced by adding isopropyl-β-d-thiogalactopyranoside to a final concentration of 1 mM, and the induced cultures were incubated at 18°C for 36 hours with 200 rpm shaking. Cells were harvested by centrifugation at 4000*g* for 15 min in 50 ml of aliquots, and the pellets were stored at −20°C.

### Fungal culture and sample preparation

Fifty plates of the ice nucleation active fungal species *M. alpina* or *E. parvispora* were grown on full-strength potato dextrose agar plates (VWR International GmbH) and malt yeast extract agar plates (Difco Malt Extract Broth, Thermo Fisher Scientific, USA), respectively. Growth occurred at room temperature for 2 weeks and then at 4°C for about 4 weeks. Pure water was obtained from Millipore Milli-Q Integral 3 water purification system (Merck Chemicals GmbH), autoclaved at 121°C for 15 min, and filtered through a 0.1-μm bottle top filtration unit (VWR International GmbH). For the droplet freezing experiments, solutions of fungal mycelium were prepared as described previously with the following modifications. The fungal mycelium of five agar plates was collected in a sterile 50-ml tube, and the weight of the mycelium was determined gravimetrically. Aliquots of 50 ml of pure water were added to the mycelium. The samples were vortexed three times at 2700 rpm for 1 min. The mycelial washes for all experiments were filtered through a 0.1-μm bottle-top filtration unit (VWR International GmbH), and the resulting mycelial wash contained INs from spores and mycelial surfaces. For filtration experiments, the 0.1-μm filtrate was filtered through 50-kDa Molecular Weight Cut-Off Polyethersulfone (MWCO PES) ultrafiltration centrifugation units (Thermo Fisher Scientific), and the IN concentration was determined by Twin-plate Ice Nucleation Assay (TINA) measurements. To assess ice nucleation activity in the bacteria harboring His-SUMO-Ent1_pET-16b or His-SUMO-Mor1_pET-16b, the harvested pellets were thawed on ice. One milligram of pellet was thawed on ice and suspended in 1 ml of pure water. A glass tissue grinder (MilliporeSigma, Merck KGaA, Darmstadt, Germany) was used to manually disrupt the cells. The suspension was centrifuged at 14,000*g* for 30 min at 4°C to pellet the cell debris, and the supernatant was measured using TINA. ClearColi cells prepared by the same procedure served as a negative control. Likewise, yeast cells were measured in pure water, and the yeast not expressing INpros served as a negative control.

### Purification of INpros from *E. parvispora*

The mycelial wash of *E. parvispora* was lyophilized and reconstituted in pure water to ~9 mg/ml for analysis by fast protein liquid chromatography using a HiPrep 16/60 Sephacryl S-100 HR column (16 mm by 600 mm) (Cytiva, USA). The elution buffer was 50 mM sodium phosphate and 100 mM sodium chloride (pH 7.5). The flow rate was 0.5 ml/min with absorbance recorded at 280 nm. Fractions were collected in 1 ml of volumes. For SDS–polyacrylamide gel electrophoresis (PAGE), aliquots of the size exclusion fractions were mixed with a fourth volume of Bolt lithium dodecyl sulfate (LDS) Sample Buffer and a 10th volume of NuPAGE Sample Reducing Agent (Thermo Fisher Scientific) and heated at 95°C for 10 min. Samples were loaded onto a NuPAGE Bis-Tris Mini Protein gel, 4 to 12% next to a molecular weight marker (PageRuler Prestained Protein Ladder, catalog no. 26616, Thermo Fisher Scientific). The electrophoresis setting was a constant voltage of 150 V for 60 min. Staining was performed using a Pierce Silver Stain Kit (Thermo Fisher Scientific).

### TINA experiments

Ice nucleation experiments were performed using the high-throughput TINA, which has been described in detail elsewhere (*39*). In a typical experiment, the investigated IN sample was serially diluted 10-fold by a liquid handling station (epMotion ep5073, Eppendorf). Then, 96 droplets (3 μl) per dilution were placed on two 384-well plates and tested with a continuous cooling rate of 1°C/min from 0° to −30°C with a temperature uncertainty of ±0.2°C. The droplet freezing was determined by two infrared cameras (Seek Therman Compact XR, Seek Thermal Inc.). For each experiment, the obtained fraction of frozen droplets (*f*_ice_) and the counting error were used to calculate the cumulative number of IN (*N*_m_) with the associated error using Vali’s formula and Gaussian error propagation (*57*). All experiments were performed at least three times. Background freezing of pure water in our system occurred at ~−23°C. We find that independent samples from individual fungal cultures show similar results with minor variations, consistent with previous studies (*23*, *39*).

### Identification of the ice-nucleating subpopulations through HUB analysis

The HUB method (*40*) was used for the identification and quantification of the subpopulations that constitute the experimental cumulative freezing spectra. This method uses a stochastic optimization technique to extract the underlying distribution of heterogeneous ice nucleation temperatures *P*_u_ (*T*) that describes the characteristic freezing temperatures of all INs in a sample. For this, the HUB-backward code available as a Python code (https://github.com/Molinero-Group/underlying-distribution) was used to compute the differential freezing spectra *n*_m_ (*T*), representing *P*_u_ (*T*), from the cumulative freezing spectra *N*_m_ (*T*) obtained from TINA experiments. *P*_u_ (*T*) is assumed to be a linear combination of normalized Gaussian distribution functions *P*_i_ (*T*) that represent a distinct number of subpopulations *p* of the weights ci that give $\sum_{i=1}^{p}\text{ci}=1$. Each subpopulation *Pi* (*T*) is characterized by its characteristic freezing temperature mode *T*_mode,*i*_ and the spread of the temperature distribution *s_i_*. The experimentally obtained *N*_m_ (*T*) is interpolated through a spline and smoothed with a Savitzky-Golay filter of first-order polynomial with a default value of 3 for the length of the filter window. The mean squared error (MSE) defines the accuracy of the determined set of parameters for the distribution function. For analysis, optimized results with the lowest MSE were selected.

### Prediction of the protein structures with AlphaFold3

AlphaFold3 was used for structure predictions (*37*). The best-ranked (i.e., with the overall highest pLDDT scores) AlphaFold monomer models predict INpro β helix with a slight twist. The AlphaFold predictions of INpro dimers and multimeric aggregates exhibited twisted and kinked structures that do not match the observed high ice nucleation temperatures. All models were constructed using the same settings (version = 2, modelSeeds = 123).

### Prediction of the ice nucleation temperatures of INpro aggregates

The HINT algorithm applies CNT to predict the heterogeneous ice nucleation temperature (*T*_het_) on finite surfaces from experimentally determined thermodynamic and kinetic properties of water and the binding free energy of the ice-nucleating particle to ice (Δγ_bind_) (*21*). The IN surfaces of *En*INpro and *Mo*INpro are modeled as rectangles of width *W* = 3.4 nm and lengths *L* = 19 nm (*En*INpro) and 25.3 nm (*Mo*INpro), consistent with AlphaFold3 predictions. Functional aggregates of *n* INpros are assumed to have widths *W*_n_ = *n* × 3.4 nm and the same lengths, consistent with the assembly model proposed here.

The algorithm computes the minimum free energy path Δ*G* for nucleating ice on the protein surface and its subsequent growth into the solution as a function of nucleus volume. From the Δ*G* profile, it identifies the free energy barriers and calculates nucleation rates. Δ*G* is evaluated using experimental thermodynamic data for water, assuming that the fungal INpros—same as the bacterial INpro (*21*)—bind ice as strongly as ice itself (Δγ_bind_ = −2 γ_ice–liquid_) and have a line tension τ = 10 pN for the ice-protein-water three-phase line. Two nucleation barriers, ΔG* (*T*), emerge from the analysis: one for ice growth on the protein surface and another for growth into bulk water (*21*). The larger barrier limits nucleation and sets *T*_het_. To determine *T*_het_, Δ*G* profiles are calculated every 0.1°C from the melting point to the homogeneous nucleation temperature, and the largest barrier at each temperature is used to compute the heterogeneous nucleation rate. We report as *T*_het_ the temperature for which the nucleation frequency is 10^2^ s^−1^, corresponding to crystallization at a cooling rate of 1°C min^−1^.
