# Supplementary material for: A previously unrecognized class of fungal ice-nucleating proteins with bacterial ancestry
Source: Sci Adv. 2026 Mar 11;12(11):eaed9652. doi: 10.1126/sciadv.aed9652 (PMC12978214; doi:10.1126/sciadv.aed9652)
Supplement: Supplementary file 1 — Figs. S1 to S16 Legend for table S1 Tables S2 to S5 [file sciadv.aed9652_sm.pdf]

Science Advances
AAAS

Supplementary Materials for

A previously unrecognized class of fungal ice-nucleating proteins with bacterial ancestry

Rosemary J. Eufemio *et al.*

Corresponding author: Konrad Meister, konradmeister@boisestate.edu

Sci. Adv. **12**, eaed9652 (2026)
DOI: 10.1126/sciadv.aed9652

The PDF file includes:

Figs. S1 to S16
Legend for table S1
Tables S2 to S5

Other Supplementary Material for this manuscript includes the following:

Table S1

Supplementary Figures

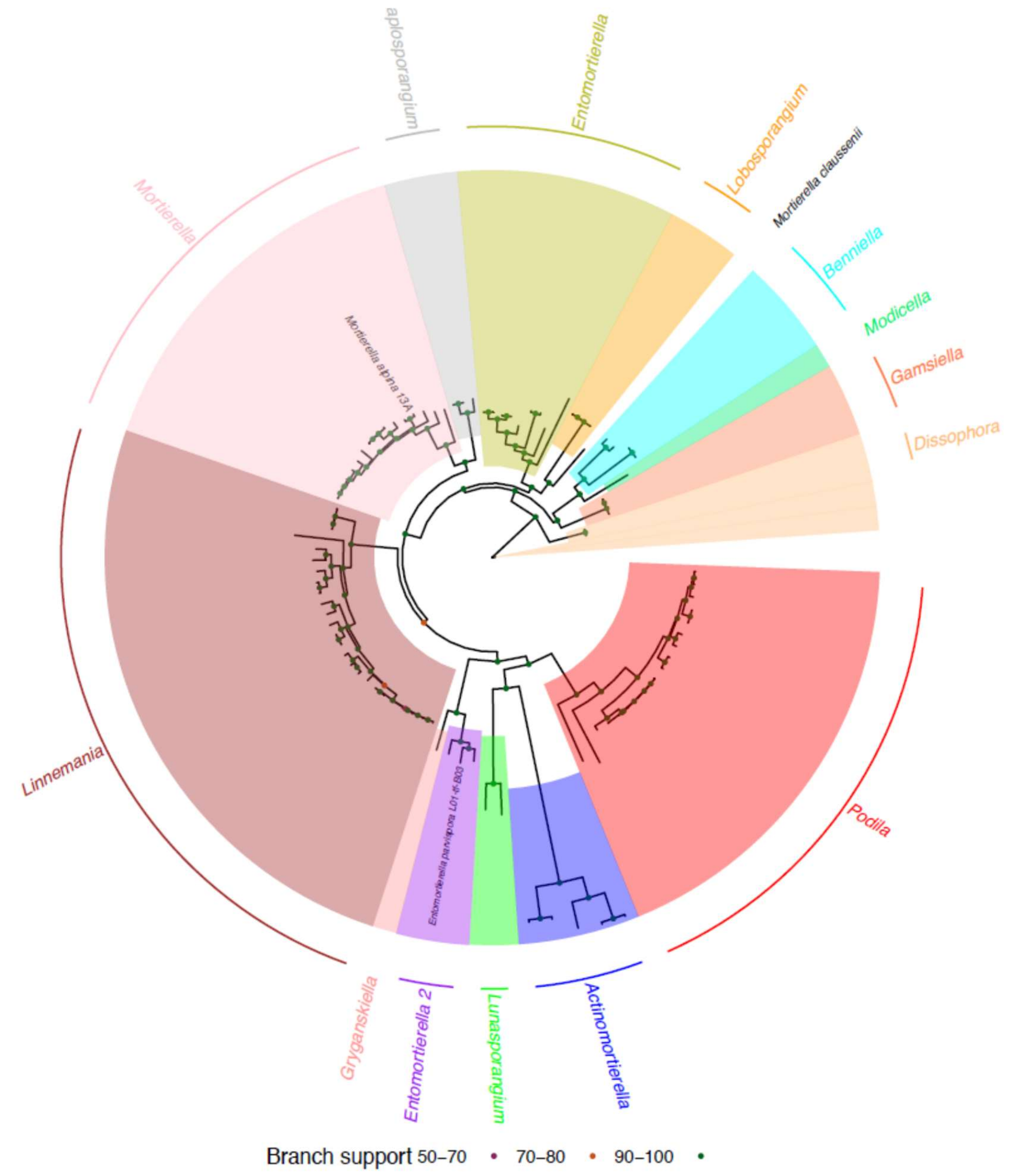

Fig. S1. Phylogenetic tree of *Mortierellaceae* genomes used to determine the identity of L01-tf-B03.

The core genome tree was built using genomes from previously defined species for *Mortierellaceae (27)*. Newly sequenced genomes are annotated at the tips with their strain names.

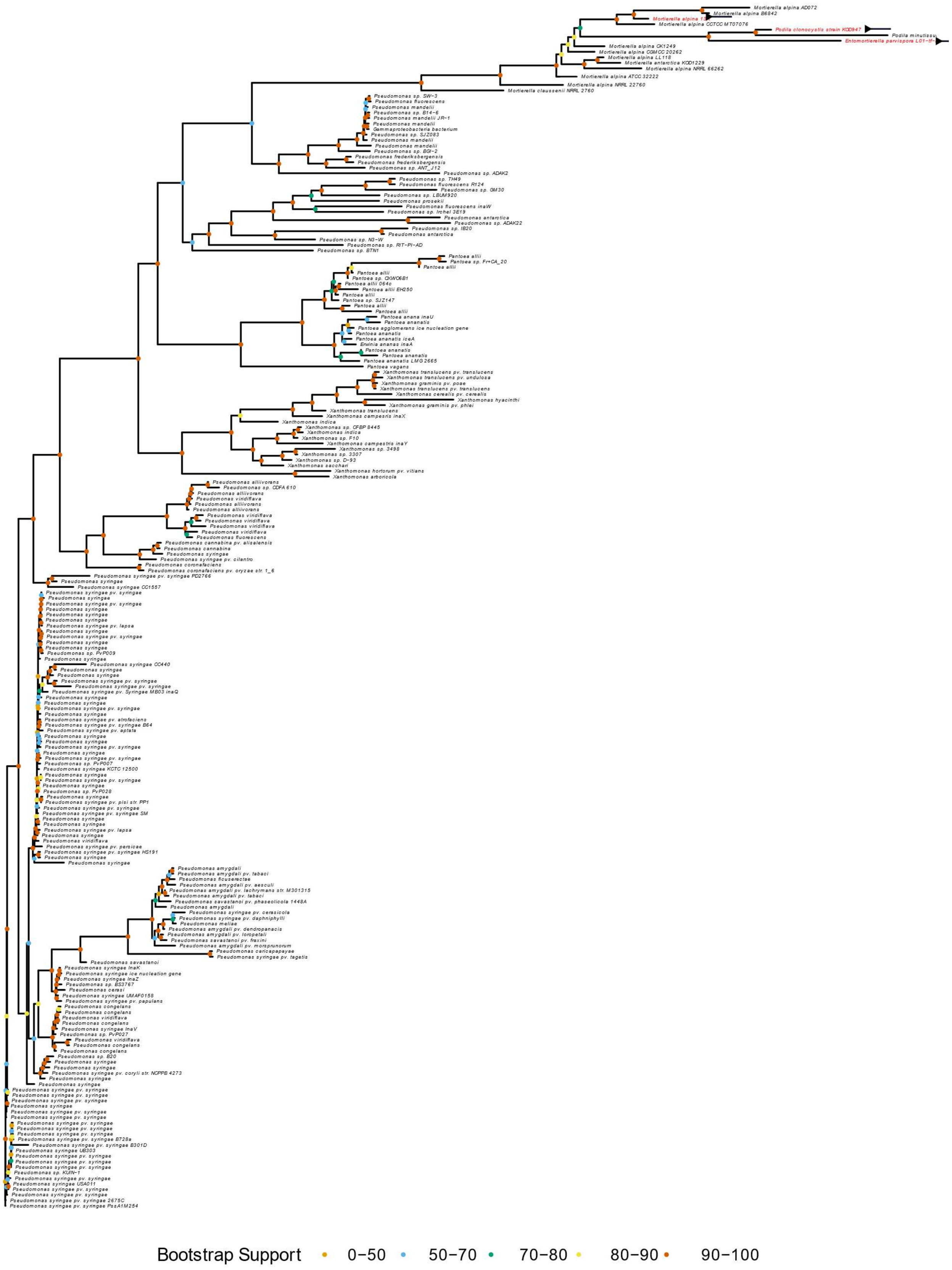
Bootstrap Support
0−50
50−70
70−80
80−90
90−100

Fig. S2. Phylogenetic relationship between fungal and bacterial ice-nucleating protein genes. Shown is a maximum likelihood phylogenetic tree illustrating the evolutionary placement of the genes coding for INpros in the *Mortierellaceae* relative to bacterial *InaZ* orthologues from *Pantoea*, *Pseudomonas*, and *Xanthomonas*. The location of the clade of fungal genes flanking the *Pseudomonas mandelii* clade suggests a horizontal gene transfer from a *P. mandelii* ancestor to the *Mortierellaceae*. Bootstrap values are shown at the nodes.

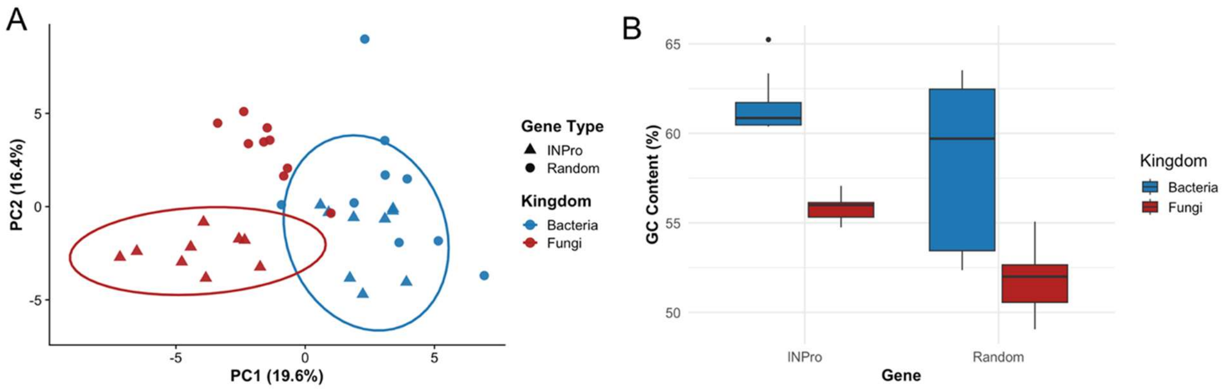

Fig. S3. **Comparative analysis of GC content in bacterial and fungal genes.**
(**A**) Principal component analysis based on relative synonymous codon usage data. Three cluster are evident: a single cluster of random bacterial genes and bacterial INpro genes, a cluster of random fungal genes, and a cluster fungal INpro genes. The single cluster of bacterial Inpro and random bacterial genes is in line with a bacterial origin of the bacterial INpro genes while the clear distinction between random fungal genes and the fungal Inpro genes is in line with an independent, probably bacterial, evolutionary origin of fungal INpro genes compared to random fungal genes. (**B**) Comparison of GC content between random bacterial and fungal genes and bacterial and fungal INpro genes. Statistical analysis indicates that bacterial INpro genes have a mean GC content of 61.58%, compared to 58.77% for random genes, resulting in a mean difference of 2.81% (95% Confidence interval: [-0.87, 6.5], (Welch's two sample t-test, p-value: 0.1195), which is not statistically significant. In contrast, fungal INpro genes exhibit a mean GC content of 55.86%, significantly higher than the 51.88% observed in random genes, with a mean difference of 3.98% (95% CI: [2.36, 5.60], p-value: 0.00026), indicating a statistically significant difference. Additionally, when comparing bacterial INpro genes to fungal INpro genes, the mean GC content for bacteria is 61.58%, while for fungi it is 55.86%, resulting in a mean difference of 5.72% (95% CI: [4.38, 7.05], p-value: 1.30E-06), which is also statistically significant. The absence of a statistical difference in GC content between random bacterial genes and bacterial INpro genes is in line with a bacterial origin of the bacterial INpro genes while the significantly higher GC content of fungal INpro genes (and thus more similar to bacterial random and INpro genes) compared to random fungal genes is in line with a bacterial origin of the fungal INpro genes.

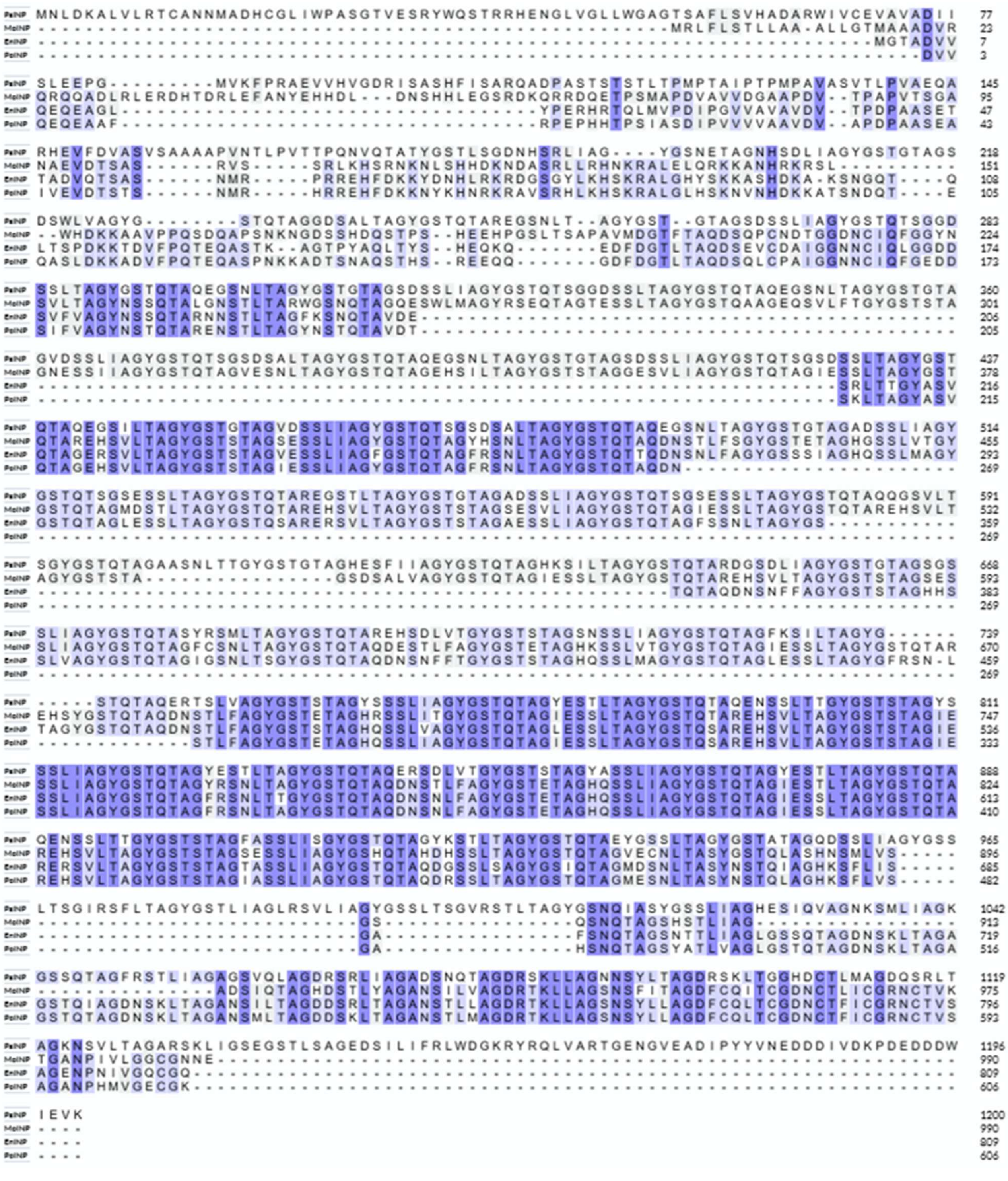

Fig. S4. Sequence alignment of *Ps*INpro, *Mo*INpro, *En*INpro, and *Po*INpro.
Residues with 100% conservation across all four INpros are highlighted in dark purple, while residues conserved in three of the four sequences are shown in light purple. Alignment was performed using the UniProt Align Tool.

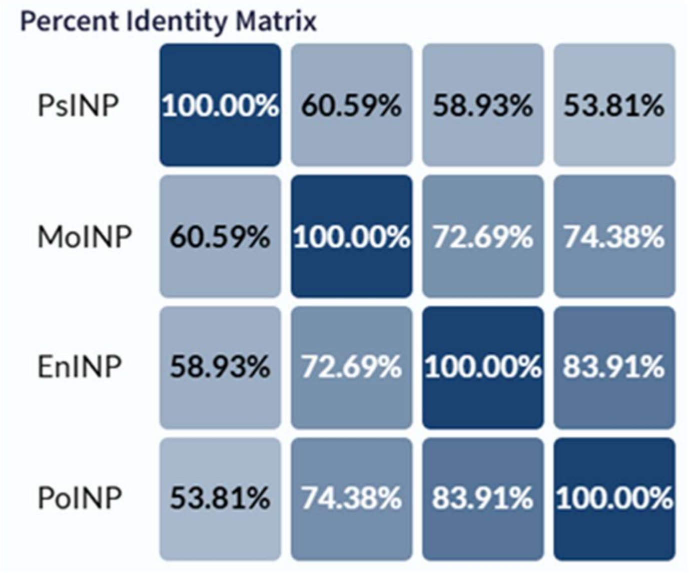

Fig. S5. Percent identity matrix of *Ps*INpro, *Mo*INpro, *En*INpro, and *Po*INpro.
The matrix displays pairwise sequence identity scores, calculated as the percentage of identical residues relative to the total alignment length.

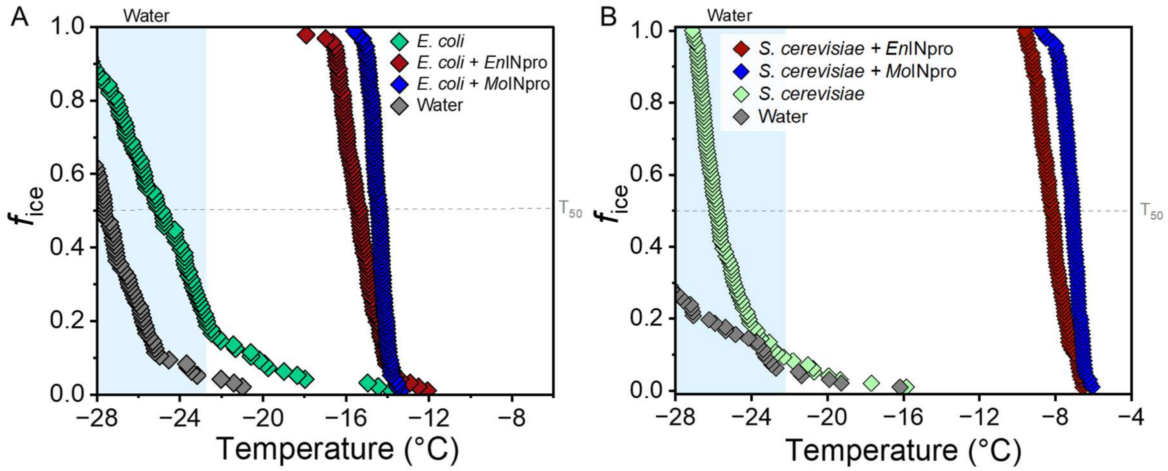

Fig. S6. Ice nucleation activity of bacteria and yeast expressing the fungal INpros.
The bacteria *E. coli* (**A**), water, and the yeast *S. cerevisiae* (**B**) show no ice nucleation activity. Upon expressing *Mo*INpro or *En*INpro both show potent ice nucleation activity. These results provide genetic evidence that the *InaZ* orthologues encoding *Mo*INpro and *En*INpro are sufficient to confer ice nucleation activity. The reduced activity of the expressed fungal INpros in *E. coli* might result from low protein expression level, partial misfolding, or different post-translational modifications.

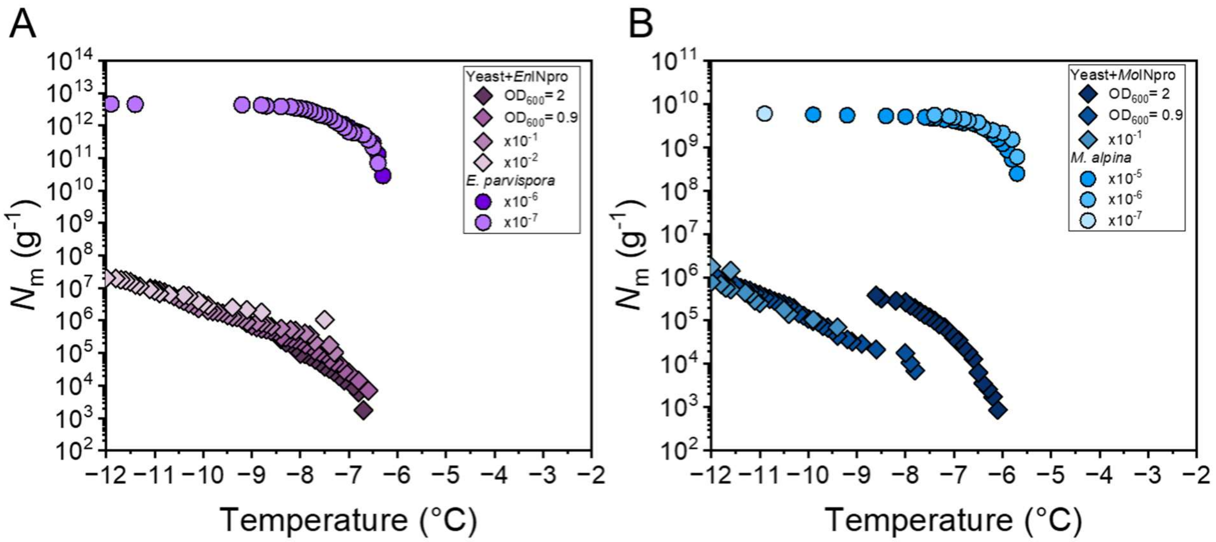

Fig. S7. Cumulative freezing spectra for serial dilutions of *S. cerevisiae* expressing (A) *En*INpro and (B) *Mo*INpro.

Shown alongside are serial 10-fold dilutions of the respective native host samples (initial concentration 1 mg/mL, see Fig. 5). Yeast samples with OD_{600}=0.9, equaling ~1 mg/mL which were diluted serially. The ice nucleation activity of *En*INpro and *Mo*INpro expressing yeast closely matches that of the native host dilutions associated with the lowest active subpopulations identified by the HUB analysis (see Fig. 5). The freezing temperatures differ by no more than ±0.4 °C between heterologous and native samples, showing that INpro expression is sufficient to confer similar ice nucleation activity.

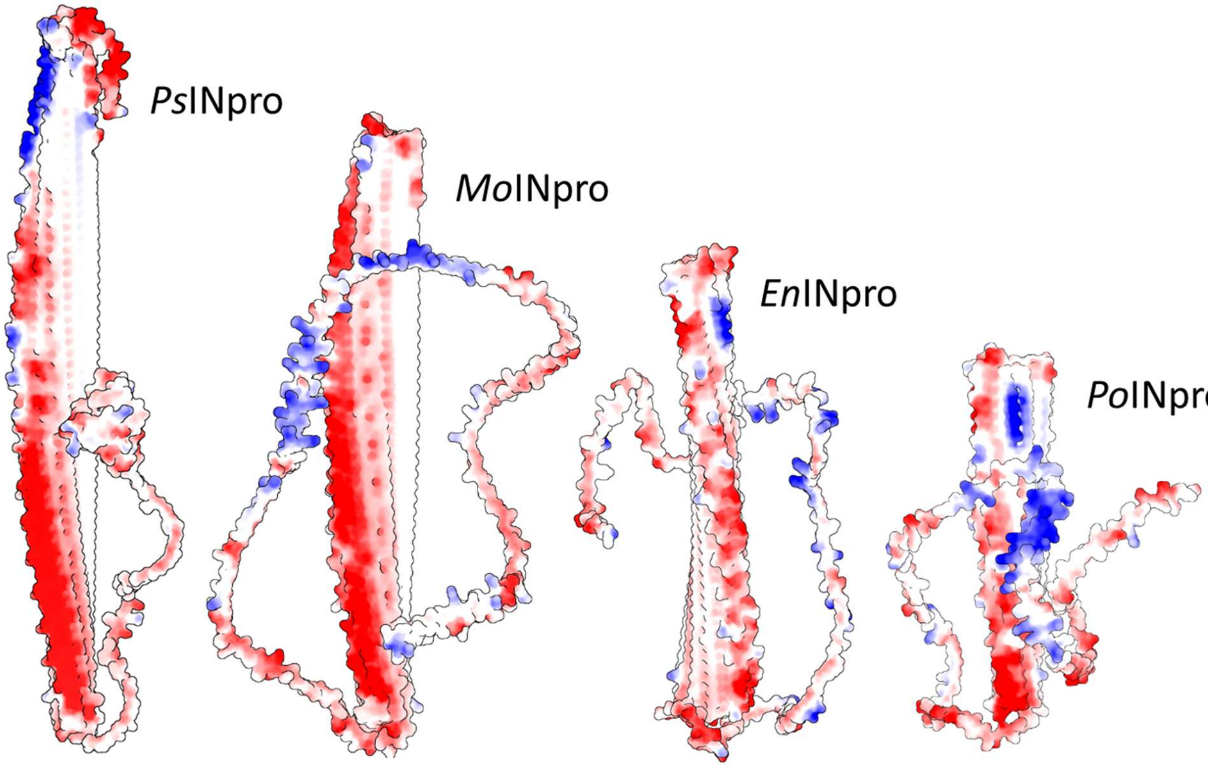

Fig. S8. Electrostatic surface maps of bacterial INpro (*Ps*INpro) and fungal INpros (*Mo*INpro, *En*INpro, and *Po*INpro) highlighting differences in charge distribution. Positively charged residues (Lysine, Arginine, and Histidine) are shown in shades of blue, while negatively charged residues (Aspartic Acid and Glutamic Acid) are shown in red, respectively. In *Ps*INpro, positively charged residues cluster near the C-terminus, while negatively charged residues are found near the N-terminus along one side of the solenoid. In contrast, fungal INpros exhibit a more alternating charge arrangement along the solenoid, with a notable positively charged patch at the N-terminus. Additionally, fungal INpros incorporate higher histidine content, a feature largely absent in bacterial INpros. These differences suggest distinct assembly and functional mechanisms between bacterial and fungal INpros.

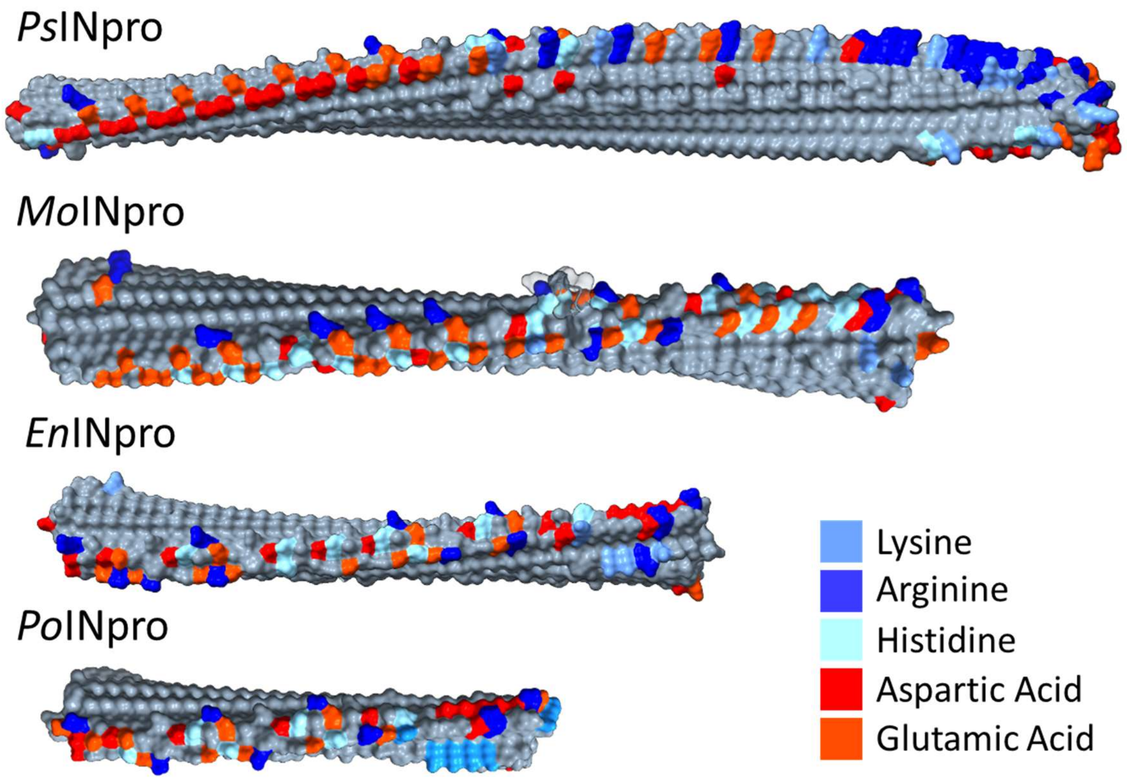

Fig. S9. Electrostatic surface maps of bacterial INpros (*Ps*INpro) and fungal INpros (*Mo*INpro, *En*INpro, and *Po*INpro) highlighting differences in charge distribution.
Positively charged residues (Lysine, Arginine, and Histidine) are shown in shades of blue, while negatively charged residues (Aspartic Acid and Glutamic Acid) are shown in red and orange, respectively. In *Ps*INpro, positively charged residues cluster near the C-terminus, while negatively charged residues are found near the N-terminus along one side of the solenoid. In contrast, fungal INpros exhibit a more alternating charge arrangement along the solenoid, with a notable positively charged patch at the N-terminus. Additionally, fungal INpros incorporate higher histidine content, a feature largely absent in bacterial INpros. These differences suggest distinct assembly and functional mechanisms between bacterial and fungal INpros.

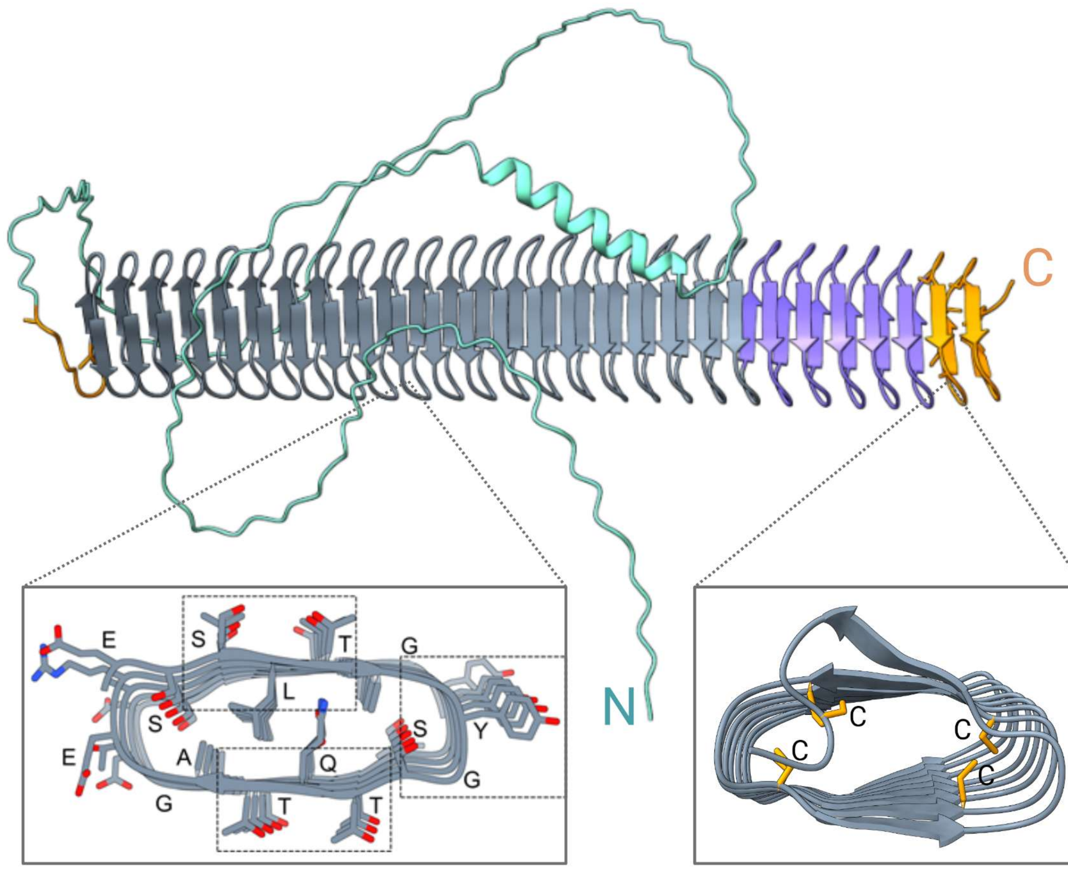

Fig. S10. AlphaFold3 model of *Po*INpro and cross-sections through the solenoid.
The model of *Po*INpro is colored by different domains with arrows representing beta strands. The model highlights a β-solenoid fold (grey, purple) adjacent to the disulfide capping motif (orange). Cross-section through the central domain region. Residues are identified by their one letter codes. Boxes indicated the location of the characteristic TQT, SLT, and YGS motifs. Cross-section of the central domain's capping structures, where adjacent cysteine residues can form disulfide bonds that stabilize the β-solenoid fold and prevent uncoiling.

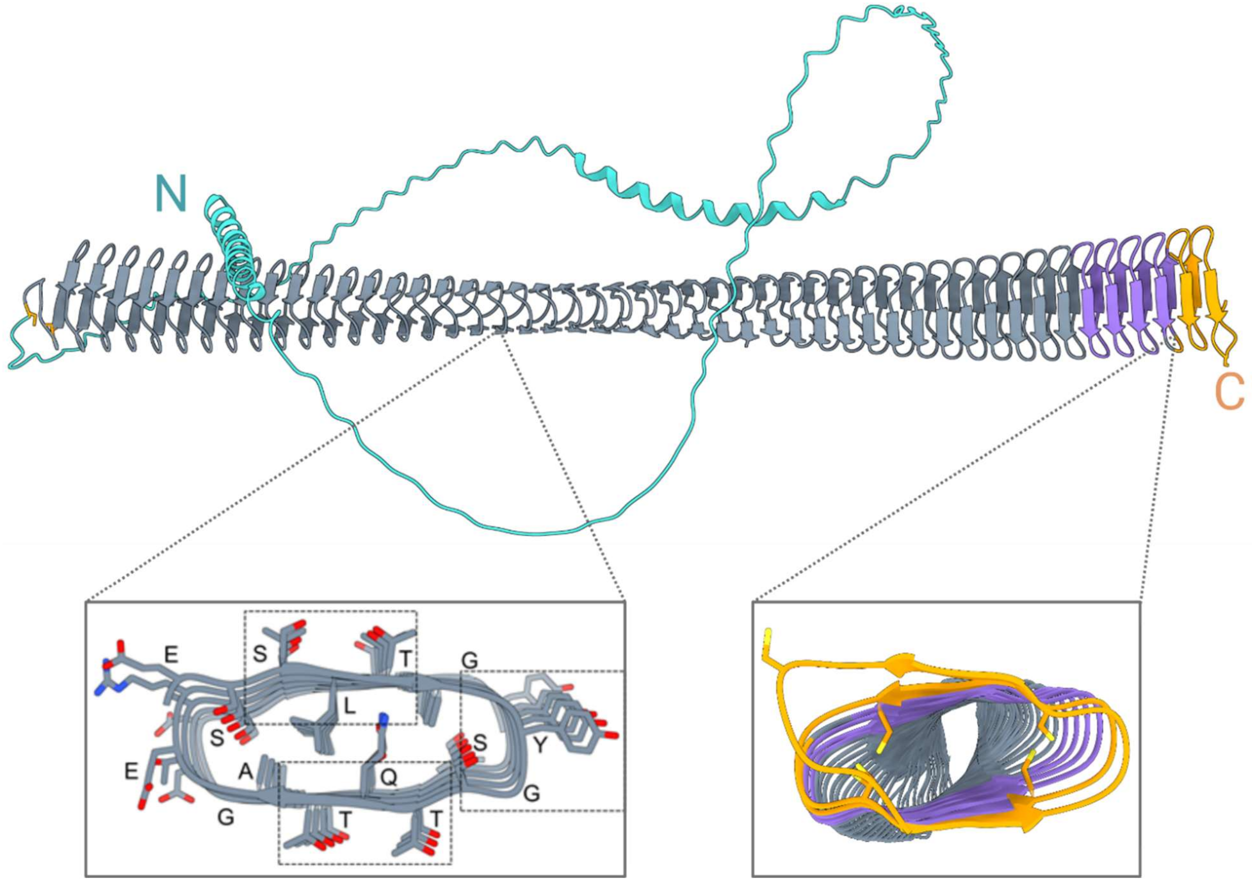

Fig. S11. AlphaFold3 model of *Mo*INpro and cross-sections through the solenoid.
The model of *Mo*INpro is colored by different domains with arrows representing beta strands. The model highlights a β-solenoid fold (grey, purple) adjacent to the disulfide capping motif (orange). Cross-section through the central domain region. Residues are identified by their one letter codes. Boxes indicated the location of the characteristic TQT, SLT, and YGS motifs. Cross-section of the central domain's capping structures, where adjacent cysteine residues can form disulfide bonds that stabilize the β-solenoid fold and prevent uncoiling.

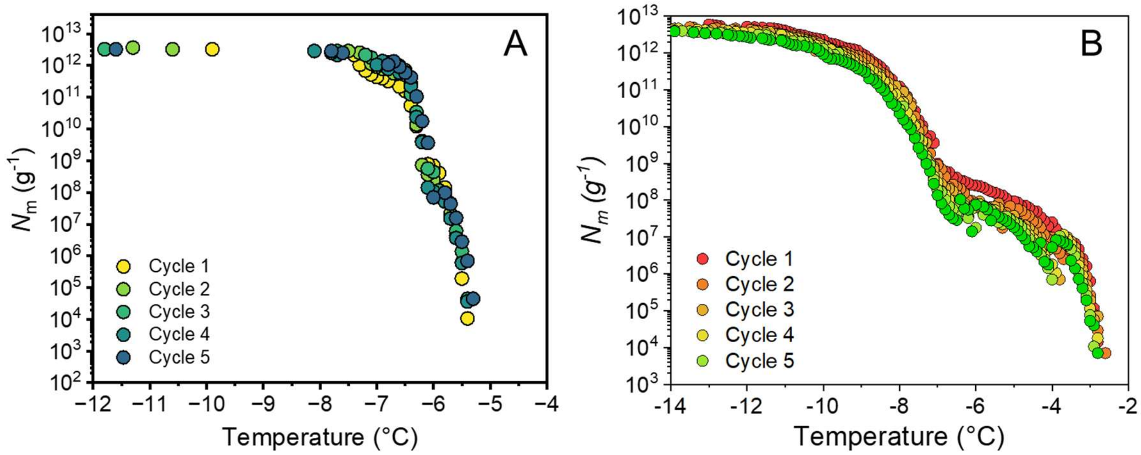

Fig. S12. Effects of freeze–thaw cycles on bacterial and fungal ice nucleation activity. Shown are the cumulative numbers of INs per unit mass (N_m) of (**A**) *E. parvispora* (1mg/mL) and (**B**) 1 mg/mL Snomax^{TM} (inactivated *P. syringae*)*(18)*. While freeze–thaw cycles do not affect the maximum freezing efficiencies of fungal INs, they reduce the maximum efficiencies observed in bacterial INs, as shown previously*(18, 26)*.

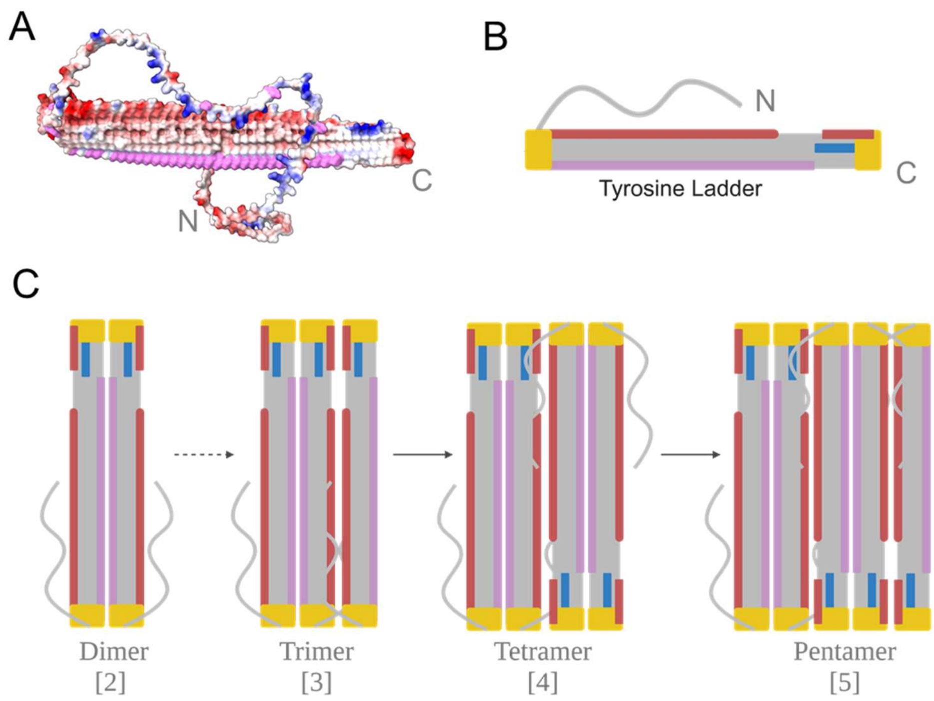

Fig. S13. Proposed INpro assembly mechanism into functional aggregates.
(**A, B**) Electrostatic surface map of the *En*INpro monomer. Positively charged residues are shown in blue, negatively charged residues are shown in red, while the tyrosine ladder is shown in violet. (**C**) INpro assembly mechanism into dimers, trimers, tetramers, and pentamers. Dimer formation is mediated by stacking of tyrosine ladders (violet areas) between adjacent monomers. The trimer, tetramer, and pentamer formation are likely enabled by electrostatic interactions between the outward-facing positively and negatively charged residues located on opposing ends of the β-solenoids, with the assistance of the charged N-terminal linker.

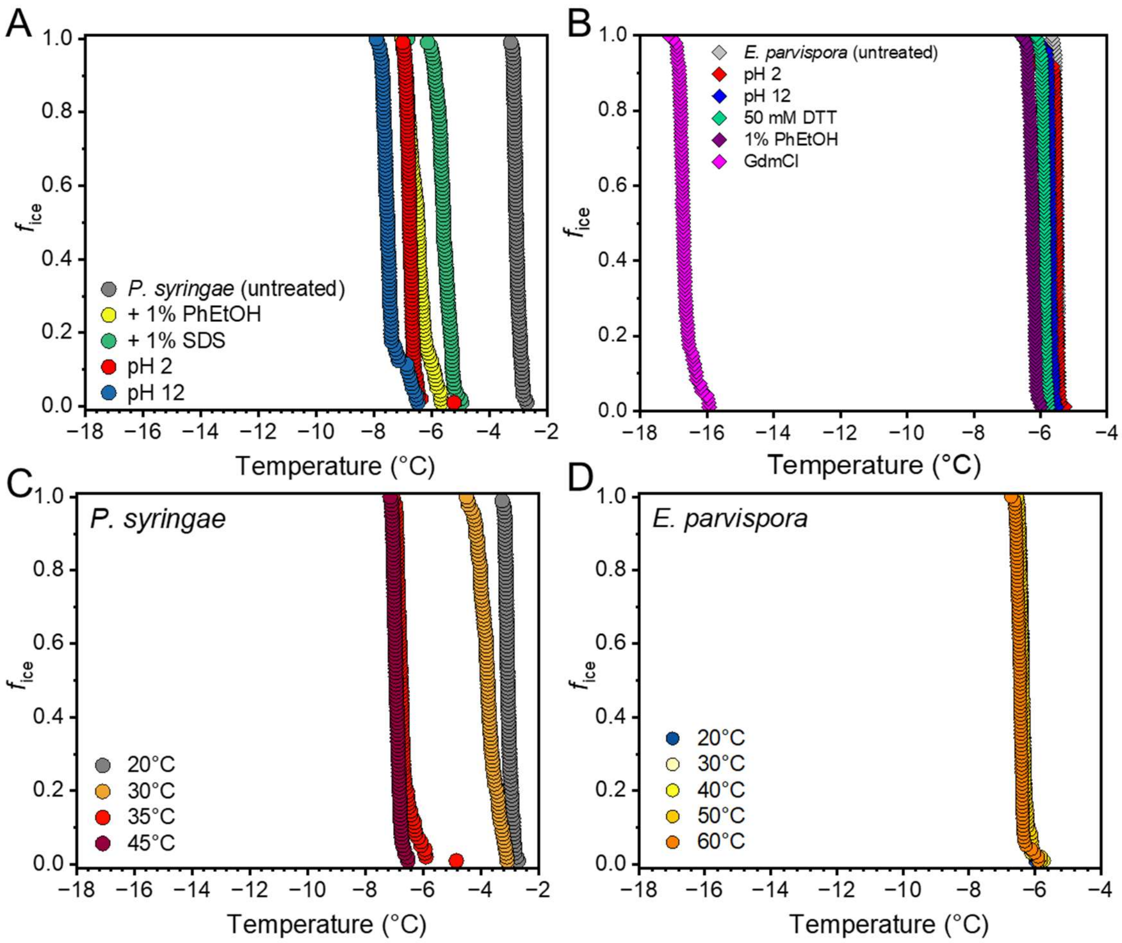

Fig. S14. Freezing experiments of aqueous solutions of *P. syringae* (A, C) and *E. parvispora* (B, D) in the presence of various cosolutes and at different temperatures.

Shown are the fraction of frozen droplets f_{ice} for solutions of *En*INpros at a fixed 0.2 mg/mL concentration. The addition of 50 mM of the disulfide bond breaker dithiothreitol (DTT, green), 1% wt of the membrane fluidizer 2-Phenylethanol (PhEtOH, purple) and 1% wt of the surfactant sodium dodecyl sulfate (SDS, yellow) has negligible effects on the freezing efficiency of *En*INpros. Additionally, *En*INpros remain active under extreme pH conditions, retaining ice nucleation activity at pH 2 (red) and pH 12 (blue). Activity is abolished in the presence of the protein denaturant guanidinium hydrochloride (GdmCl, pink), indicating the necessity of an intact protein structure for ice nucleation. In contrast, the activity of *P. syringae* is decreased by both moderate temperatures and cosolutes.

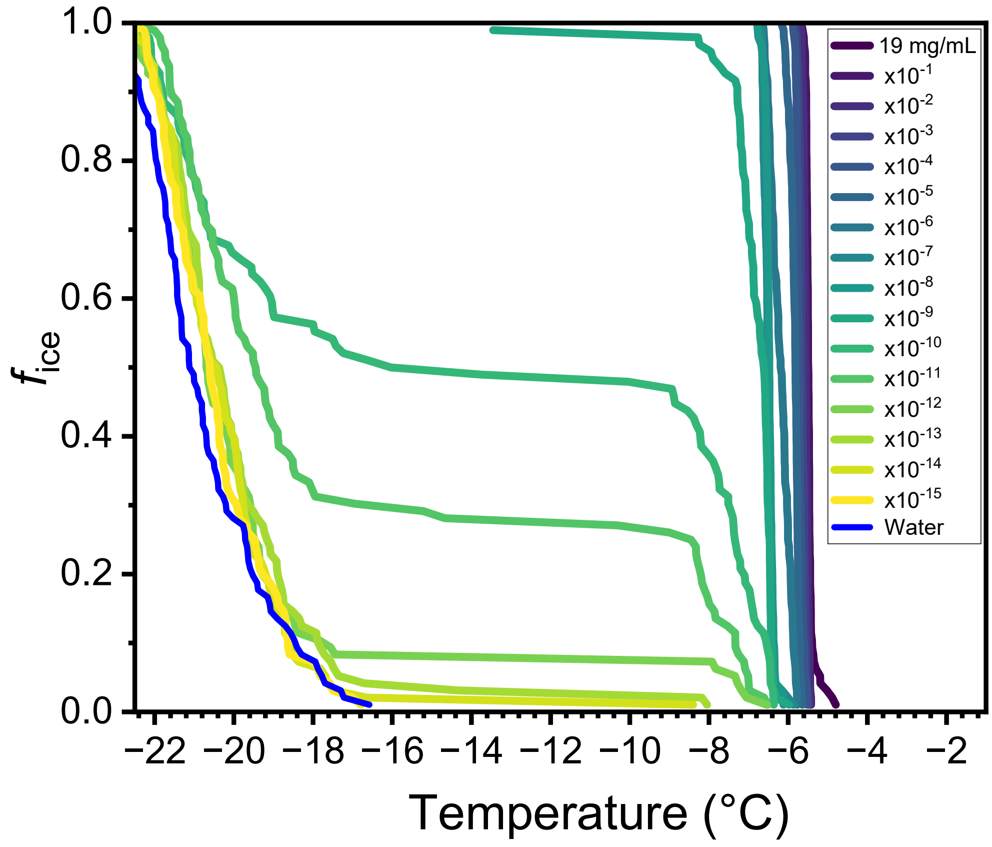

Fig. S15. Freezing experiments of aqueous solutions containing fungal INs from *E. parvispora*.

Shown are the fraction of frozen droplets (f_{ice}) vs temperature for the 10-fold dilution series of INs in pure water, with an initial concentration of 19 mg/mL. The results demonstrate that INs from *E. parvispora* retain their potent ice-nucleating activity (>-6.5 °C) even at concentrations as low as 1.9 ng/mL. Given the molecular weight of 82.8 kDa, this corresponds to picomolar concentrations, highlighting the remarkable potency of fungal INpros in ice nucleation.

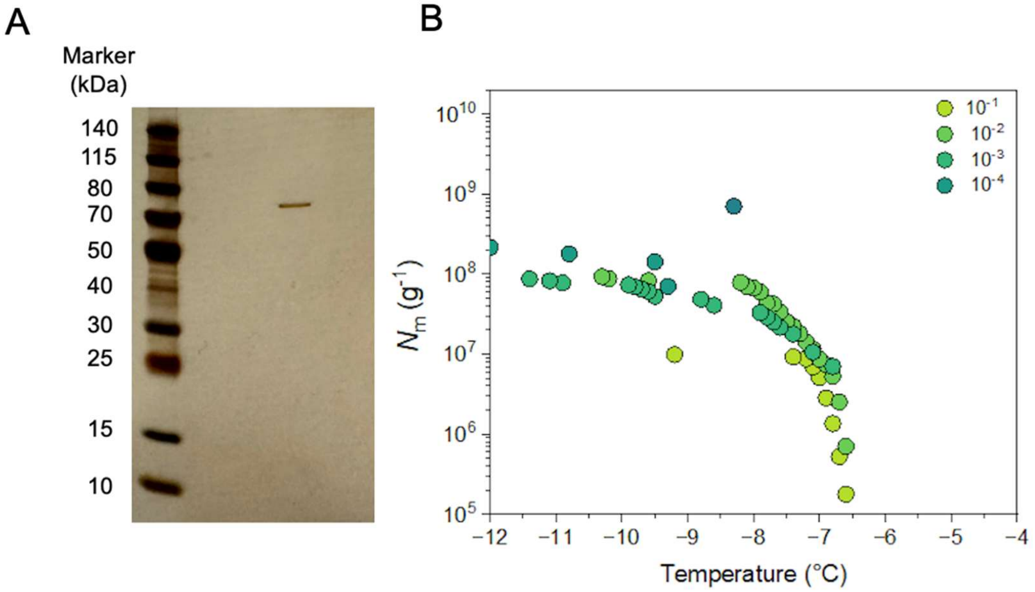

Fig. S16. Purification of aqueous solutions of INpros from *Entomortierella parvispora*. (**A**) SDS-PAGE of the mycelial wash of *E. parvispora* after purification by size exclusion shows a pure band at the expected molecular weight of ~81 kDa. (**B**) Cumulative freezing spectra confirm that purified INpros enable potent ice-nucleating activity.

Supplementary Tables

Table S1. Gene sequences associated with ice nucleation were downloaded from UniProt on February 10, 2025. List is added as an additional file due to the large size.

Table S2. Parameter of the normalized distribution functions of the freezing spectra of *M. alpina* obtained through the HUB method using one, two or three Gaussian subpopulations. *T*mode is the mode (maximum) of the Gaussian distribution of heterogeneous ice nucleation temperatures of the corresponding subpopulation, *s* is the spread of the distribution of the subpopulations, and *c* the fraction of the subpopulation. The mean squared error (MSE) describes the quality of the found optimized solution for the cumulative spectra obtained with the HUB analysis relative to the experimental cumulative spectra.

Number of Populations	MSE (%)	$T_{mode,1}$ (°C)	s_1 (°C)	c_1	$T_{mode,2}$ (°C)	s_2 (°C)	c_2	$T_{mode,3}$ (°C)	s_3	c_3
1	1.68	-6.18	0.28	1.0	-	-	-	-	-	-
2	0.62	-5.90	0.18	0.68	-7.54	0.64	0.32	-	-	-
3	0.63	-5.79	0.14	0.38	-6.48	0.32	0.59	-6.71	0.53	0.03

Table S3. Parameter of the normalized distribution functions of the freezing spectra of *E. parvispora* obtained through the HUB method using one, two or three Gaussian subpopulations.

*T*mode is the mode (maximum) of the Gaussian distribution of heterogeneous ice nucleation temperatures of the corresponding subpopulation, *s* is the spread of the distribution of the subpopulations, and *c* the fraction of the subpopulation. The mean squared error (MSE) describes the quality of the found optimized solution for the cumulative spectra obtained with the HUB analysis relative to the experimental cumulative spectra.

Number of Populations	MSE (%)	$T_{mode,1}$ (°C)	s_1 (°C)	c_1	$T_{mode,2}$ (°C)	s_2 (°C)	c_2	$T_{mode,3}$ (°C)	s_3	c_3
1	2.38	-7.03	0.35	1.0	-	-	-	-	-	-
2	1.30	-5.66	0.1	$9.8\text{x}10^{-5}$	-6.92	0.28	0.99	-	-	-
3	0.36	-5.61	$9.2\text{x}10^{-2}$	$8.4\text{x}10^{-5}$	-6.51	0.17	0.24	-7.51	0.35	0.76

Table S4. Predicted ice nucleation temperatures as a function of the number of protein monomers in the *Mo*INpro and *En*INpro aggregates.

The ice nucleation temperatures are predicted by classical nucleation theory using the HINT algorithm and are presented as a function of the number of INpros, *NINpro*, from *M. alpina* and *E. parvispora*. The analysis considers rectangular surfaces formed by parallel-aligned INpro monomers, assuming a monomer with ice-binding surface of width of 3.4 nm and length of 25.3 nm for *M. alpina* and 19 nm for *E. parvispora*, as predicted by Alphafold3.

Width	Number of INPs	$T_{het\mathit{En}INP}$	$T_{het\mathit{Mo}INP}$
3.4	1	257.9	258.2
6.8	2	263.9	264.2
10.2	3	265.9	266.5
13.6	4	266.9	267.6
17	5	267.5	268.3
20.4	6	268.1	268.7
27.2	8	268.6	269.3
40.8	12	269.3	269.9
54.4	16	269.5	270.2

Table S5. *Fusarium* genomes sequenced and analyzed in this study.
For strains previously reported to exhibit ice nucleation activity, supporting references from the literature are provided.

Genus	Strain	Ice Nucleation Activity	Sequence
Fusarium acuminatum	*3-68*	Confirmed*(10)*	sequenced by us
Fusarium avenaceum	*F156N33*	Confirmed*(25)*	sequenced by us
