# Supplementary material for: A previously unrecognized class of fungal ice-nucleating proteins with bacterial ancestry
Source: Sci Adv. 2026 Mar 11;12(11):eaed9652. doi: 10.1126/sciadv.aed9652 (PMC12978214; doi:10.1126/sciadv.aed9652)
Supplement: Supplementary file 2 — Table S1 [file sciadv.aed9652_table_s1.zip › aed9652_table_s1.pdf]

Accession	Contig or G	Start	End	Strand	Organism
AF013159.	AF013159.	.	.	.	Pseudomonas syringae InaK
AF387802.	AF387802.	.	.	.	Pantoea ananatis iceA
AJ001086.	AJ001086.	.	.	.	Pseudomonas syringae InaV
CP073636.	CP073636.	1695945	1699823	+	Pseudomonas syringae InaZ
CP090356.	CP090356.	1982222	1986574	+	Pantoea ananatis
CP125958.	CP125958.	3968795	3973147	+	Pantoea allii EH250
CP126314.	CP126314.	3918975	3923183	.	Pantoea allii 064c
D14992.1	D14992.1	.	.	.	Pantoea anana inaU
EU360731.	EU360731.	.	.	.	Pseudomonas syringae pv. Syringae MB03 ina
GCA_0005	AZCI01000	5181	7920	+	Mortierella alpina B6842
GCA_0010	LDAW0100	5742	6342	+	Mortierella alpina CCTC M207067
GCA_0149	JACLYT010	12325	13084	+	Podila minutissima UFMGCB 3722
GCA_0156	JAAAUT010	25	1099	-	Mortierella alpina AD072
GCA_0156	JAABKD010	4336	4957	+	Mortierella alpina NRRL 66262
GCA_0158	JAAAHY010	8	983	+	Mortierella alpina CK1429
GCA_0158	JAAAIB010	2	767	-	Mortierella antarctica KOD1229
GCA_0188	JAHJDA010	133807	137020	+	Gammaproteobacteria bacterium
GCA_0197	JAIFTL0100	7624	9019	-	Mortierella alpina LL118
GCA_0227	JAABNM01	5088	6093	+	Mortierella claussenii NRRL 2760
GCA_0230	JALIGY010	3024217	3026572	+	Mortierella alpina CBS 899.72
GCA_0239	JALIRG010	886330	889018	-	Mortierella alpina ATCC 32222
GCA_0294	JANATF010	274999	277294	-	Mortierella alpina CGMCC 20262
GCF_00001	NC_00577	1852449	1855812	+	Pseudomonas savastanoi pv. phaseolicola 144
GCF_00001	NC_00700	1807673	1811717	+	Pseudomonas syringae pv. syringae B728a
GCF_0001	NZ_CP031	1296691	1300387	+	Pseudomonas amygdali pv. lachrymans str. M
GCF_0001	NZ_CP046	3742925	3746597	-	Pseudomonas coronafaciens pv. oryzae str. 1_
GCF_0001	NZ_ADGB0	54338	55439	-	Pseudomonas syringae pv. syringae 642
GCF_0002	NZ_CP005	1287973	1291186	+	Pseudomonas mandelii JR-1
GCF_0002	NZ_AKJP02	71363	74060	+	Pseudomonas sp. GM30
GCF_0002	NZ_CM001	2796002	2798237	+	Pseudomonas fluorescens R124
GCF_0003	NZ_CM001	5082243	5084451	-	Pseudomonas syringae pv. syringae B64
GCF_00041	NZ_CM001	1826379	1830405	+	Pseudomonas syringae pv. syringae SM
GCF_0004	NZ_CP034	1850086	1854100	+	Pseudomonas syringae pv. pisi str. PP1
GCF_0004	NZ_AVCR0	19660	23428	+	Pseudomonas syringae pv. syringae 1212
GCF_0004	NZ_CP045	1639610	1643660	+	Pseudomonas syringae USA011
GCF_0004	NZ_CP047	1659002	1663058	+	Pseudomonas syringae UB303
GCF_0004	NZ_CP093	1778593	1781710	+	Pseudomonas syringae CC440
GCF_0004	NZ_CP007	4045101	4049148	-	Pseudomonas syringae CC1557
GCF_0004	NZ_BATH0	71	779	-	Pantoea ananatis DAR76143
GCF_0005	NZ_AYTM0	4211046	4214970	+	Pseudomonas syringae KCTC 12500
GCF_00071	NZ_JMJJ01	69635	72149	+	Pantoea ananatis LMG 2665
GCF_0008	NZ_CM003	713427	716511	-	Xanthomonas cerealis pv. cerealis
GCF_0009	NZ_JYHD01	2302	2926	+	Pseudomonas syringae pv. daphniphylli
GCF_0009	NZ_JYHE01	102	2583	+	Pseudomonas meliae
GCF_0009	NZ_JYHG01	11198	13940	-	Pseudomonas amygdali pv. dendropanacis
GCF_0009	NZ_JYHJ01	782133	786171	-	Pseudomonas syringae pv. syringae

GCF_00097	NZ_AWQP(	52638	56520	-	Pseudomonas syringae pv. coryli str. NCPPB 4
GCF_00098	NZ_CP0062	4261074	4262985	-	Pseudomonas syringae pv. syringae HS191
GCF_00098	NZ_CP0059	1702155	1706040	+	Pseudomonas syringae pv. syringae B301D
GCF_00128	NZ_CP0059	5324735	5328647	+	Pseudomonas syringae UMAF0158
GCF_00128	NZ_CXOJ01	87332	90953	-	Xanthomonas graminis pv. phlei
GCF_00129	NZ_LGLN01	85500	87633	-	Pseudomonas syringae pv. cilantro
GCF_00140	NZ_LJPW01	103169	105029	-	Pseudomonas caricapapayae
GCF_00146	NZ_LKEM0	2	926	-	Pseudomonas syringae pv. syringae PD2766
GCF_00148	NZ_CP0131	1675793	1679813	+	Pseudomonas syringae pv. lapsa
GCF_00162	NZ_LAGV01	62842	66874	-	Pseudomonas syringae pv. syringae
GCF_00164	NZ_LWLR0	1290915	1294803	+	Pantoea sp. OXWO6B1
GCF_00164	NZ_CP0156	3748651	3752311	-	Pseudomonas antarctica
GCF_00167	NZ_LYUP01	135270	137199	-	Pseudomonas syringae pv. syringae
GCF_00190	NZ_MPBM(	15	1773	+	Pseudomonas sp. BTN1
GCF_00225	NZ_NMOJ0	47578	50791	+	Pseudomonas mandelii
GCF_00226	NZ_NIAW0	53856	56352	+	Pseudomonas savastanoi pv. fraxini
GCF_00230	NZ_NTMH(	587	3023	+	Pantoea allii
GCF_00230	NZ_NTMH(	311580	312621	+	Pantoea allii
GCF_00269	NZ_LKBW0	48	3123	+	Pseudomonas amygdali
GCF_00272	NZ_LKCA01	152762	156557	+	Pseudomonas viridiflava
GCF_00290	NZ_CP0265	2024897	2027072	+	Pseudomonas amygdali pv. morsprunorum
GCF_00290	NZ_CP0265	1683829	1687879	+	Pseudomonas syringae pv. syringae
GCF_00290	NZ_MLEM(	255781	259801	+	Pseudomonas syringae pv. syringae
GCF_00290	NZ_MLES01	246351	250407	+	Pseudomonas syringae pv. syringae
GCF_00291	NZ_NBAQ0	55750	59728	-	Pseudomonas syringae pv. syringae
GCF_00291	NZ_NBAL01	819683	823565	-	Pseudomonas syringae pv. syringae
GCF_00291	NZ_NBAJ01	22036	25930	+	Pseudomonas syringae
GCF_00291	NZ_MLEX0	75774	79818	-	Pseudomonas syringae pv. syringae 2675C
GCF_00304	NZ_CP0284	2641813	2645833	-	Pseudomonas syringae pv. atrofaciens
GCF_00305	NZ_CP0097	2049534	2053191	+	Xanthomonas translucens pv. undulosa
GCF_00314	NZ_QGHF0	171562	175213	-	Pantoea allii
GCF_00341	NZ_QPBY01	21688	22510	+	Pseudomonas syringae pv. syringae
GCF_00341	NZ_QPBU0	372	2433	+	Pseudomonas syringae pv. syringae
GCF_00341	NZ_QPBR0	0	1074	-	Pseudomonas syringae pv. syringae
GCF_00341	NZ_QPBP0	2	1328	-	Pseudomonas syringae pv. syringae
GCF_00341	NZ_QPBP0	1653	2937	-	Pseudomonas syringae pv. syringae
GCF_00341	NZ_QPCE01	55413	56283	-	Pseudomonas syringae pv. syringae
GCF_00341	NZ_QPDY0	66611	67535	-	Pseudomonas amygdali pv. tabaci
GCF_00373	NZ_MOBQ(	100651	103306	-	Pseudomonas frederiksbergensis
GCF_00373	NZ_MOBR(	64120	66715	-	Pseudomonas frederiksbergensis
GCF_00385	NZ_CP0277	3864601	3868450	-	Pseudomonas sp. LBUM920
GCF_00431	NZ_SISB01(	38925	39684	+	Pseudomonas sp. BGI-2
GCF_00782	NZ_VIUF01	158497	161329	-	Pseudomonas sp. SJZ083
GCF_00782	NZ_VIVB01	273976	277486	+	Pantoea sp. SJZ147
GCF_00837	NZ_VOBN0	166574	169406	+	Pseudomonas sp. ANT_J12
GCF_00917	NZ_AP0203	1736973	1741029	+	Pseudomonas sp. KUIN-1
GCF_00969	NZ_RHGD0	5497507	5500720	-	Pseudomonas mandelii

GCF_0097(	NZ_CP0461	2997961	2998915	-	Pseudomonas sp. IB20
GCF_0097(	NZ_CP0434	198241	201307	-	Xanthomonas hyacinthi
GCF_0117(	NZ_CP0502	1185627	1189443	-	Pseudomonas coronafaciens
GCF_0129:	NZ_CP0528	1643936	1647500	-	Pseudomonas sp. ADAK22
GCF_0129:	NZ_CP0528	7004509	7008010	-	Pseudomonas sp. ADAK2
GCF_0129{	NZ_JAAQX\	357909	360978	+	Pseudomonas mandelii
GCF_0132(	NZ_JABLUS	0	1386	+	Pantoea allii
GCF_0132(	NZ_CP053{	226803	230016	-	Pseudomonas sp. B14-6
GCF_0141!	NZ_JACHO(	68794	70105	+	Xanthomonas sp. 3307
GCF_0141!	NZ_JACHOI	67779	70923	+	Xanthomonas sp. 3498
GCF_0142(	NZ_JACHNI	1288038	1291695	+	Xanthomonas sp. F10
GCF_0163(	NZ_JAEILI0	144847	146770	-	Pseudomonas syringae
GCF_0163(	NZ_JAEIKN(	117137	119345	-	Pseudomonas syringae
GCF_0163(	NZ_JAEIKL(	55666	59548	-	Pseudomonas syringae
GCF_0163(	NZ_JAEIKF(	56176	59914	-	Pseudomonas syringae
GCF_0163(	NZ_JAEIKC(	207952	211027	-	Pseudomonas syringae
GCF_0163(	NZ_JAEIJZ0	185	2825	+	Pseudomonas syringae
GCF_0163(	NZ_JAEIJY0	96	2736	+	Pseudomonas syringae
GCF_0163(	NZ_JAEIJV0	1	2161	+	Pseudomonas syringae
GCF_0163(	NZ_JAEIJW	1	718	-	Pseudomonas syringae
GCF_0163(	NZ_JAEIJO(	2	815	-	Pseudomonas syringae
GCF_0165!	NZ_CP067(	1912427	1913387	+	Pseudomonas cannabina pv. alisalensis
GCF_0166:	NZ_CP067(	2435637	2438517	-	Pseudomonas sp. SW-3
GCF_0166!	NZ_JAEKFL(	1307878	1311631	-	Pseudomonas sp. TH49
GCF_0166!	NZ_CP068(	1863366	1867398	+	Pseudomonas syringae
GCF_0173(	NZ_CP064(	1214404	1218082	+	Xanthomonas translucens pv. translucens
GCF_0173(	NZ_CP064(	3564275	3566525	-	Xanthomonas translucens pv. translucens
GCF_0177/	NZ_JAGHW	397	1600	+	Xanthomonas sp. D-93
GCF_0178:	NZ_JAFFZW	648399	652194	+	Pseudomonas alliivorans
GCF_0178:	NZ_JAFFZV	549831	553626	+	Pseudomonas alliivorans
GCF_0178:	NZ_JAFFZU	43307	46958	-	Pseudomonas alliivorans
GCF_0178:	NZ_JAFIBF(	2836587	2840571	-	Pseudomonas sp. PvP007
GCF_0178:	NZ_JAFIBL(	5552160	5556180	-	Pseudomonas sp. PvP028
GCF_0178:	NZ_JAFIBG(	3923472	3927486	-	Pseudomonas sp. PvP009
GCF_0178:	NZ_JAFIBK(	1552261	1555855	-	Pseudomonas sp. PvP027
GCF_0179:	NZ_JABEBE	400020	402363	+	Pantoea ananatis
GCF_0183:	NZ_AP024/	4539311	4542578	-	Pseudomonas amygdali pv. tabaci
GCF_0183{	NZ_JAHAR\	382983	387003	-	Pseudomonas syringae
GCF_0183{	NZ_JAHAS/	358559	362585	-	Pseudomonas syringae
GCF_0183{	NZ_CP074/	1588265	1592297	+	Pseudomonas syringae
GCF_0183{	NZ_CP074/	1569304	1573324	+	Pseudomonas syringae
GCF_0183{	NZ_CP074/	1741164	1745196	+	Pseudomonas syringae
GCF_0183!	NZ_CP074!	1620536	1624592	+	Pseudomonas syringae
GCF_0185:	NZ_CP053(	1657007	1660082	+	Pseudomonas congelans
GCF_0185:	NZ_CP053(	1783351	1787341	+	Pseudomonas congelans
GCF_0186/	NZ_JAHCZ(	21756	25506	+	Pseudomonas syringae pv. aptata
GCF_0191(	NZ_CP077;	3242663	3246554	-	Pseudomonas viridiflava

GCF_0191(	NZ_CP0777	4661888	4665683	+	Pseudomonas viridiflava
GCF_0191(	NZ_CP0777	2157070	2161009	+	Pseudomonas viridiflava
GCF_0191(	NZ_CP0777	2161457	2165108	+	Pseudomonas viridiflava
GCF_0192S	NZ_JAHVXY	213382	217126	-	Pantoea allii
GCF_0202(	NZ_CP0842	1755067	1758334	+	Pseudomonas amygdali
GCF_0205	NZ_JAHPJG	87063	90963	-	Pseudomonas sp. B20
GCF_0209	NZ_CP0766	1679884	1683562	+	Pseudomonas savastanoi
GCF_0211	NZ_JAGTPN	185327	188882	-	Pseudomonas sp. CDFA 610
GCF_0216(	NZ_WKDS(	56707	58780	-	Pseudomonas congelans
GCF_0216(	NZ_WJYR0	55971	59703	-	Pseudomonas syringae
GCF_0218	NZ_WKCZ0	97487	99041	+	Pseudomonas syringae
GCF_0219(	NZ_CP0743	1715939	1719473	+	Xanthomonas translucens
GCF_0219(	NZ_CP0762	3188553	3192231	-	Xanthomonas graminis pv. poae
GCF_0225	NZ_JAKMV	346788	350844	+	Pseudomonas syringae pv. syringae
GCF_0225	NZ_JAKMV	234768	235569	+	Pseudomonas syringae pv. lapsa
GCF_0225	NZ_JAKMV	275215	279259	-	Pseudomonas syringae pv. syringae
GCF_0225	NZ_JAKMV	239608	243652	+	Pseudomonas syringae pv. syringae
GCF_0225	NZ_JAKMV	128383	131458	-	Pseudomonas syringae pv. Syringae PssA1M2
GCF_0225	NZ_JAKMV	730001	734045	+	Pseudomonas syringae pv. syringae
GCF_0225	NZ_CP092	1724890	1728253	+	Pseudomonas syringae pv. tagetis
GCF_0225	NZ_JAKZM	409531	411829	+	Pantoea allii
GCF_0226	NZ_JAKJPQ	2	698	-	Xanthomonas indica
GCF_0226	NZ_JAKLXA	730614	734415	+	Xanthomonas indica
GCF_0232(	NZ_CP0892	1865135	1867754	+	Pseudomonas amygdali pv. loropetali
GCF_0232	NZ_CP0662	1676318	1680368	+	Pseudomonas syringae pv. syringae
GCF_0232	NZ_CP0662	3616786	3619960	-	Pseudomonas syringae
GCF_0235(	NZ_JAMDH	138492	142368	-	Pseudomonas syringae
GCF_0239	NZ_JAMXF	351082	354877	+	Pseudomonas alliivorans
GCF_0242	NZ_CP100	2114653	2115754	+	Xanthomonas sacchari
GCF_0245	NZ_JALNPN	510820	514309	+	Pantoea sp. Fr+CA_20
GCF_0245	NZ_CP085	1984532	1988087	+	Pseudomonas ficuserectae
GCF_0249	NZ_CP103	2466468	2470170	+	Pseudomonas sp. N3-W
GCF_0256	NZ_JANZK	24321	26805	+	Pseudomonas sp. RIT-PI-AD
GCF_0256	NZ_CP102	1833340	1836997	+	Xanthomonas sp. CFBP 8445
GCF_0259	NZ_JAPDC(	271438	272473	-	Pantoea ananatis
GCF_0262	NZ_CP110	1713521	1717259	+	Pseudomonas syringae
GCF_0262	NZ_CP110	1668915	1672935	+	Pseudomonas syringae
GCF_0262	NZ_JAPJYL	22774	23974	-	Pantoea vagans
GCF_0284	NZ_JAGSO	247267	251149	+	Pseudomonas syringae pv. syringae
GCF_0284	NZ_JAGSO	55976	59870	-	Pseudomonas syringae pv. syringae
GCF_0293	NZ_JARNIY	66000	70074	-	Pseudomonas syringae pv. syringae
GCF_0293	NZ_JARNIX	392480	396530	+	Pseudomonas syringae pv. syringae
GCF_0293	NZ_JARNJB	55854	59886	-	Pseudomonas syringae
GCF_0294	NZ_JAROC	75290	79172	-	Pseudomonas syringae
GCF_0298	NZ_CP123	6044194	6046444	+	Pseudomonas amygdali pv. aesculi
GCF_0298	NZ_JAFFRZ	4574402	4578278	-	Pseudomonas syringae pv. papulans
GCF_9000	NZ_LT2223	1617290	1619705	+	Pseudomonas cerasi

GCF_90009	NZ_FNCX01	763047	766983	+	Pseudomonas sp. BS3767
GCF_90010	NZ_FNKU0	781354	785293	+	Pseudomonas cannabina
GCF_90010	NZ_FNJH01	55817	59549	-	Pseudomonas congelans
GCF_90010	NZ_LT6297	4066050	4069947	+	Pseudomonas prosekii
GCF_90010	NZ_LT6297	6794399	6797468	+	Pseudomonas mandelii
GCF_90011	NZ_FOPR01	55793	59525	-	Pseudomonas syringae
GCF_90011	NZ_FOQB0	55941	59961	-	Pseudomonas syringae
GCF_90011	NZ_FOTU01	61140	65079	-	Pseudomonas syringae
GCF_90011	NZ_FOVV01	316191	320223	+	Pseudomonas syringae
GCF_90018	NZ_FYDT01	169002	172851	-	Pseudomonas sp. Irchel 3E19
GCF_90023	NZ_ODAM0	14629	18523	+	Pseudomonas syringae pv. persicae
GCF_90023	NZ_LT9624	1687554	1691580	+	Pseudomonas syringae pv. syringae
GCF_90028	NZ_OLMP0	2895315	2897661	-	Pseudomonas syringae pv. cerasicola
GCF_90058	NZ_UTUR0	361200	365214	+	Pseudomonas viridiflava
GCF_90058	NZ_UUIL01	688633	692671	+	Pseudomonas viridiflava
GCF_90059	NZ_UVTT01	643671	647403	-	Pseudomonas viridiflava
GCF_90062	NZ_UYXQ0	56	1181	-	Pseudomonas antarctica
GCF_90062	NZ_UYXQ0	10258	12331	-	Pseudomonas antarctica
GCF_90249	NZ_CABVH	103547	107054	+	Pseudomonas fluorescens
GCF_90249	NZ_CABVIK	361758	364590	-	Pseudomonas fluorescens
HG992337.	HG992337.	.	.	.	Xanthomonas arboricola
JAAAIF000	JAAAIF010	6803	8683	+	Podila clonocystis strain KOD947
KY817116.	KY817116.	.	.	.	Xanthomonas campestris inaY
TBD	g6506	.	.	.	Entomortierella parvispora L01-tf-B03
LR828257.	LR828257.	.	.	.	Xanthomonas hortorum pv. vitians
M26382.1	M26382.1	.	.	.	Pantoea agglomerans ice nucleation gene
TBD	g9171	.	.	.	Mortierella alpina 13A
X03035.1	X03035.1	.	.	.	Pseudomonas syringae InaZ
X04501.1	X04501.1	.	.	.	Pseudomonas fluorescens inaW
X17316.1	X17316.1	.	.	.	Erwinia ananas inaA
X52970.1	X52970.1	.	.	.	Xanthomonas campesris inaX
